# αE-catenin inhibits YAP/TAZ activity to regulate signalling centre formation during tooth development

**DOI:** 10.1038/ncomms12133

**Published:** 2016-07-13

**Authors:** Chun-Ying Li, Jimmy Hu, Hongbing Lu, Jing Lan, Wei Du, Nicole Galicia, Ophir D. Klein

**Affiliations:** 1 The Research Center and Department of Pathology, Zhongshan Hospital of Dalian University, Dalian 116001, China; 2 Department of Orofacial Sciences and Program in Craniofacial Biology, University of California, San Francisco, California 94143, USA; 3 Department of Pediatrics and Institute for Human Genetics, University of California, San Francisco, California 94143, USA

## Abstract

Embryonic signalling centres are specialized clusters of non-proliferating cells that direct the development of many organs. However, the mechanisms that establish these essential structures in mammals are not well understood. Here we report, using the murine incisor as a model, that αE-catenin is essential for inhibiting nuclear YAP localization and cell proliferation. This function of αE-catenin is required for formation of the tooth signalling centre, the enamel knot (EK), which maintains dental mesenchymal condensation and epithelial invagination. EK formation depends primarily on the signalling function of αE-catenin through YAP and its homologue TAZ, as opposed to its adhesive function, and combined deletion of *Yap* and *Taz* rescues the EK defects caused by loss of αE-catenin. These findings point to a developmental mechanism by which αE-catenin restricts YAP/TAZ activity to establish a group of non-dividing and specialized cells that constitute a signalling centre.

## Abstract (web summary)

It is unclear which signals regulate the establishment of the tooth signalling centre, the enamel knot (EK). Here, the authors show that EK formation depends on αE-catenin, which acts to restrict the Hippo pathway transcription factors Yes-associated protein (YAP) and TAZ.

## Graphical abstract

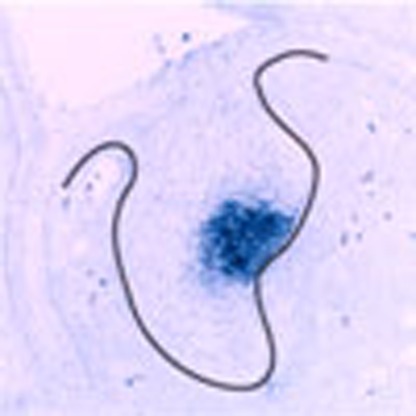

Epithelial–mesenchymal signalling regulates many developmental processes, including the outgrowth of limbs^1^, the generation of hair follicles and feathers, the budding and branching of developing kidneys, lungs and mammary glands, and the formation of teeth^2^,^3^. Tooth development, which has long served as a model for understanding epithelial–mesenchymal signalling^4^, initiates as the oral epithelium thickenss to become a multilayered structure, known as the placode (Fig. 1a). During the transition to the bud stage, the placode first invaginates into the underlying mesenchyme and then undergoes marked morphological changes that result in the formation of two epithelial protrusions at the distal end of the tooth germ, resembling a cap. Concurrently, dental mesenchyme condenses around the epithelium in response to signals from the epithelium. A key event at the bud stage is the formation of a structure called the enamel knot (EK)^5^. In mouse, only one EK is formed in incisors, whereas both primary and secondary EKs are formed in molars.

The EK is composed of a group of densely packed post-mitotic epithelial cells that express the cyclin-dependent kinase inhibitor p21 (p21^Cip1/WAF1^)^5^,^6^. The principal function of the EK is secretion of an array of signalling molecules, including Sonic hedgehog (SHH), Fibroblast growth factors (FGFs), WNTs and bone morphogenetic proteins (BMPs)^7^, which together regulate tooth morphogenesis. For example, EK-produced FGFs promote cell proliferation both in the mesenchyme and in the epithelium surrounding the EK, driving growth of the tooth germ^8^. EK-produced FGFs also induce expression of FGFs in the adjacent mesenchyme, which reciprocally signal back to the epithelium^9^, driving further development of the tooth. Thus, the EK is considered a signalling centre, and its ability to direct the behaviour of neighbouring cells parallels the function of other developmental signalling centres, such as the apical ectodermal ridge (AER) in the limb bud, the floor plate in the neural tube or the isthmus at the midbrain–hindbrain boundary. However, little is known regarding the molecular mechanism that initiates and maintains signalling centres such as the EK.

Histologically, the EK, as well as several other signalling centres such as the floor plate and the isthmus, can be identified as a group of cells with reduced mitotic index^10^,^11^, suggesting that regulated cell proliferation is crucial for their formation and function. The Hippo signalling pathway has been shown to be an important regulator of cell proliferation and differentiation^12^, although little is known about its role in tooth development. The nuclear localization of downstream transcription co-factors, Yes-associated protein (YAP) and its homologue, transcriptional coactivator with PDZ-binding motif (TAZ), allows them to bind to other transcription factors, such as TEAD1-4, and promotes expression of genes that drive cell proliferation^13^. Hippo pathway signalling or other stimuli such as mechanical signalling or increased cell density can lead to phosphorylation of YAP and TAZ by kinases, such as LATS1 and 2, thus rendering YAP and TAZ inactive through sequestration in the cytoplasm and/or degradation^14^,^15^,^16^. A central player that controls YAP/TAZ localization is α-catenin, which retains YAP/TAZ in the cytoplasm as a result of phosphorylation at the S127 site^17^, and deletion of α-catenin can result in uncontrolled YAP accumulation in the nucleus and increased transcriptional activity, driving hyperproliferation in some tissues^17^,^18^.

α-catenin is best known for its role as a component of the adherens junction, which also includes cadherins and catenins. In this capacity, α-catenins bind to actin filaments, either directly or through actin-binding proteins, to maintain cellular architecture and seal gaps between neighbouring cells^19^,^20^. In both humans and mice, three α-catenin genes have been identified, *Ctnna1*, *Ctnna2* and *Ctnna3*, which encode αE-catenin, αN-catenin and αT-catenin, respectively. αE-catenin is the most prevalent in epithelial tissues^21^ and is required specifically for the morphogenesis of ectodermal appendages. For example, deletion of *Ctnna1* with epithelial-specific K14Cre abrogates formation of the hair follicle placodes and sebaceous glands, and loss of *Ctnna1* in the mammary gland perturbs cell polarity and differentiation and inhibits alveolar epithelial expansion^22^,^23^. However, the mechanisms by which αE-catenin regulates tissue morphogenesis remain incompletely understood.

In this study, we first established that cells within the EK, which are non-proliferating and express high levels of p21, also have low levels of nuclear YAP. By genetically ablating *Ctnna1* (encoding αE-catenin) in the dental epithelium, we then showed that αE-catenin is required to prevent nuclear YAP accumulation and ectopic cell proliferation in the EK. Deletion of *Ctnna1* thus resulted in loss of the EK, epithelial invagination defects and perturbed mesenchymal condensation. Remarkably, combined deletion of *Yap* and *Taz* in the *Ctnna1* mutants was able to restore EK formation and rescue the morphogenesis phenotype. These data thus point to a mechanism by which an αE-catenin-YAP/TAZ pathway controls signalling centre formation.

## Results

### The EK has low levels of nuclear YAP

**Figure 1:**
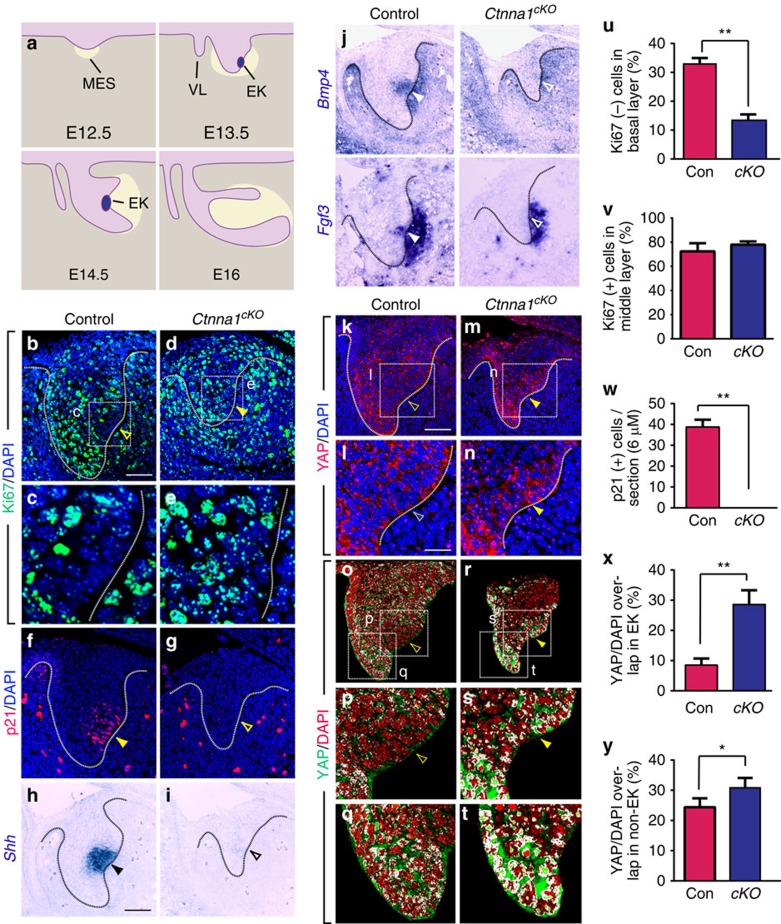
Deletion of *Ctnna1* induces YAP nuclear localization as well as cell proliferation and inhibits EK formation. (**a**) Schematic diagram of incisor morphogenesis. At E12.5, the dental epithelium (light purple) begins to invaginate into the underlying mesenchyme (MES, tan). By E13.5, the EK is formed. Subsequent epithelial-mesenchymal interactions result in mesenchymal condensation around the epithelium (yellow shaded area). At E14.5, the epithelium turns posteriorly, and the EK regresses by E16. (**b**,**c**) At E13.5, the EK consists of post-mitotic cells that are Ki67 negative (open yellow arrowhead). (**d**,**e**) Ablation of *Ctnna1* (*Ctnna1*^*cKO*^) by K14Cre results in ectopic cell proliferation in this region (yellow arrowhead in **c**). (**f**,**g**) p21 is expressed in the control EK (yellow arrowhead) but not in *Ctnna1*^*cKO*^ (compare yellow arrowhead in **f** to open yellow arrowhead in **g**). (**h**–**j**) EK markers, *Shh*, *Bmp4* and *Fgf3*, are absent in the *Ctnna1*^*cKO*^ (compare black arrowhead in **h** and white arrowheads in **j** to open black arrowhead in **i** and open white arrowheads in **j**). Mesenchymal *Bmp4* and *Fgf3* are also reduced. (**k**,**l**,**o**–**q**) At E13.5, the EK contains mostly cytoplasmic YAP (open yellow arrowheads in **k**,**l**), while strong nuclear YAP staining is detected in the surrounding epithelium (**q**). This is visualized through detection of overlapping signals (white) between YAP (green) and DAPI (red) (open yellow arrowheads in **o**,**p**). (**m**,**n**,**r**–**t**) Deletion of *Ctnna1* results in YAP nuclear localization in the presumptive EK (yellow arrowheads in **m**,**n**,**r**,**s**) and the neighbouring epithelium (**t**). (**u**,**v**) Quantification of Ki67-negative (−) cells in the basal layer (**u**) and Ki67-positive (+) cells in the area surrounding the EK (**v**) at E13.5 (mean±s.e.m., *n*=3, *P*<0.01). (**w**) Quantification of p21-positive (+) cells in control and *Ctnna1*^*cKO*^ (mean±s.e.m., *n*=3, *P*<0.01). (**x**,**y**) Quantification of nuclear YAP in the EK region (**x**) and in the protruding epithelium (**y**) in control and *Ctnna1*^*cKO*^ (mean±s.e.m., *n*=4, *P*<0.01 and 0.05 respectively). All quantifications are analysed by using Student's *t*-test. *P<0.05, **P<0.01. Dotted lines outline the epithelium of the incisor tooth germ. VL, vestibular lamina. Scale bar, 25 μm (**b**,**d**,**f**,**g**,**k**,**m**,**o**,**r**); 12 μm (**l**,**n**,**p**,**q**,**s**,**t**); 8 μm (**c**,**e**) 50 μm (**h**,**i**,**j**).

The EK is comprised of a group of post-mitotic cells, in contrast to the surrounding epithelium, where active proliferation takes place to support the continuous growth of the tissue^5^. This prompted us to ask whether proper regulation of cell proliferation is required for the formation of the EK. We focused on the incisor because it has a relatively simple structure compared with molars and contains only one EK. As previous experiments mostly investigated development of molars, we first examined cell proliferation in the incisor epithelium by means of Ki67 immunostaining at E13.5, when the formation of the EK is initiated. Similar to the primary EK in the molars, the incisor EK is marked by an area of Ki67 negative cells (Fig. 1b,c). This coincides with the expression of the cyclin-dependent kinase inhibitor p21 in the same region (Fig. 1f), indicating that EK cells are indeed no longer cycling. Importantly, *Shh*, *Bmp4* and *Fgf3*, which are expressed in the molar EK^24^,^25^,^26^, are also expressed in the incisor EK at this stage, providing us additional markers to assess for the presence of these cells (Fig. 1h,j).

To understand the molecular mechanism that induces cell cycle arrest during formation of the EK, we next examined the expression pattern of the transcription co-factor, YAP, which has been shown to be an important regulator of cell proliferation and differentiation in other tissues^12^. In particular, nuclear YAP promotes the expression of genes that are important for cell proliferation, and its sequestration in the cytoplasm counters that process. In control embryos, consistent with the proliferation profile that was observed in the EK, immunostaining showed nuclear YAP in cells that are located at the protruding lingual and labial tips of the tooth germ, where many cells are proliferating (Fig. 1k,o,q). It should be noted that cytoplasmic YAP was also present in these cells, suggesting that a basal level of regulatory control is in place to modulate the amount of YAP inside the nucleus. However, nuclear YAP expression was absent in the EK region, where YAP was mostly localized in the cytoplasm (Fig. 1k,l,o,p), consistent with an earlier observation in the molar primary EK^27^. Importantly, the expression patterns of low nuclear YAP, high levels of p21 and low levels of Ki67 observed in the incisor EK were also observed in several other known signalling centres, including the AER, floor plate and isthmus, suggesting that a common mechanism involving YAP may be employed to inhibit cell proliferation within these structures and control their formation (Supplementary Fig. 1a–x).[Fig f2][Fig f3]

### αE-catenin regulates YAP localization in the EK

The results above led us to hypothesize that the exclusion of YAP from the nucleus may be a mechanism for establishing a group of non-proliferating cells that become specialized in signal production within a growing tissue. To test this, we set out to perturb YAP localization. As cytoplasmic retention of YAP depends on the presence of αE-catenin^17^, we first asked whether absence of αE-catenin would increase nuclear YAP. We genetically deleted *Ctnna1*, which encodes αE-catenin, in the dental epithelium by crossing a conditional allele of *Ctnna1* (*Ctnna1*^*fl/fl*^) with *Keratin 14*^*Cre*^ (*K14*^*Cre*^)^28^,^29^, where Cre recombinase is regulated by the *K14* promoter and is expressed in the oral epithelium starting at E11.75, enabling deletion of αE-catenin throughout the entire tooth germ epithelium (Fig. 4a–d). Loss of *Ctnna1* resulted in YAP accumulation in the nucleus in the presumptive EK region (Fig. 1m,n,r,s). This increase in nuclear YAP paralleled what has been observed in the epidermis after deletion of αE-catenin^17^. Quantification of overlapping signals between YAP and nuclear DAPI further confirmed that αE-catenin loss led to nuclear YAP in the region that would form the EK in controls (Fig. 1x). Using Student's *t*-test, we observed a statistically significant increase of nuclear YAP in the protruding dental epithelium adjacent to the EK as well (Fig. 1t,y), suggesting that αE-catenin also inhibits YAP accumulation in these cells. Interestingly, proliferation in non-EK epithelial cells was not affected by *Ctnna1* deletion (Fig. 1v), most likely because nuclear YAP is normally present in these cells and a further increase in nuclear YAP had limited effects on cell cycle regulation.

### αE-catenin-mediated cell polarity regulates YAP localization

**Figure 2 f2:**
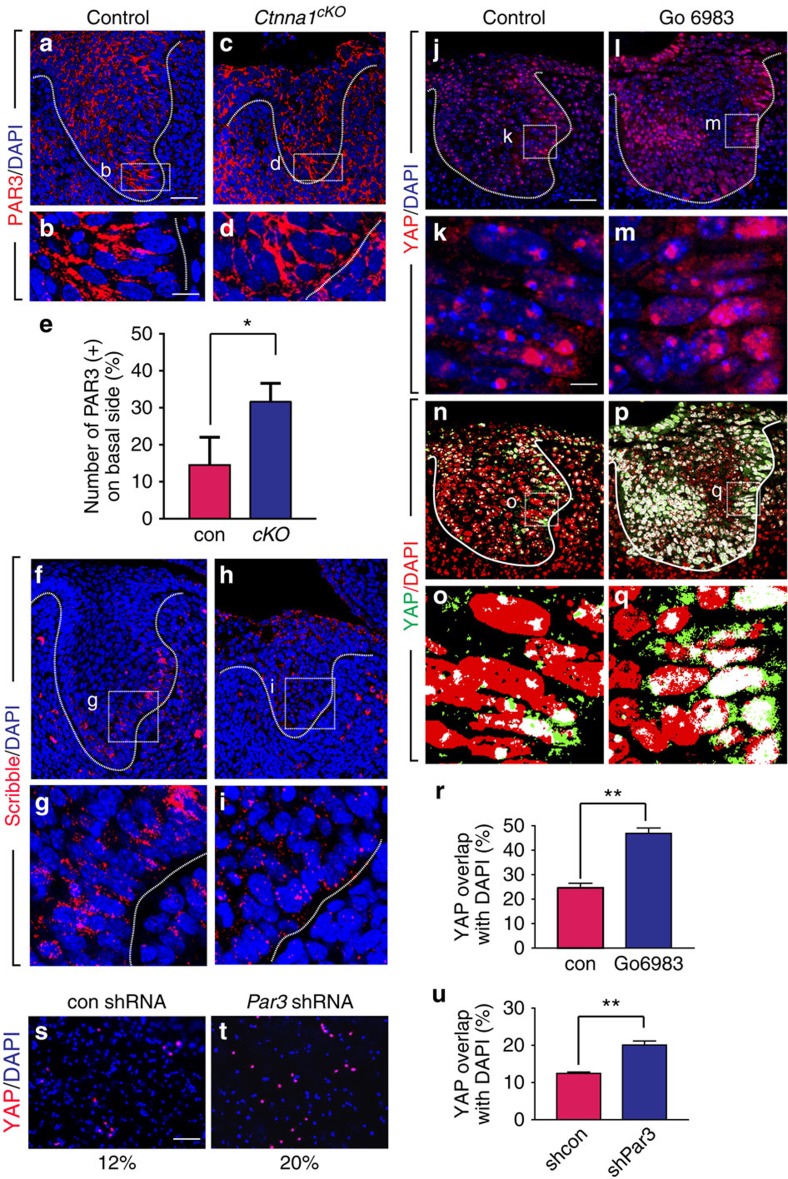
αE-catenin is required for maintaining proper cell polarity in the tooth germ epithelium. (**a**,**b**) PAR3 is apically localized in cells of the basal layer at E13.5. (**c**,**d**) Deletion of *Ctnna1* causes basal mislocalization of PAR3. (**e**) Quantification of the cells with mislocalized PAR3 in basal layers of control and *Ctnna1*^*cKO*^ tooth germs (mean±s.e.m., *n*=3, *P*<0.05). (**f**,**g**) Scribble is laterally localized in the basal layer at E13.5. (**h**,**i**) The level of Scribble protein is decreased after ablation of αE-catenin. (**j**–**q**) Inhibition of aPKC with Gö 6983 induces nuclear localization of YAP in the explant culture. (**r**) Quantification of nuclear YAP in vehicle and antagonist treated tissues (mean±s.e.m., *n*=3, *P*<0.01). (**s**,**t**) Knockdown of PAR3 with shRNA induces YAP nuclear localization in primary epithelial cells. (**u**) Quantification of nuclear YAP in control (shcon) and shRNA (shPar3)-treated cells (mean±s.e.m., *n*=3, *P*<0.01). All quantifications are analysed by using Student's *t*-test. *P<0.05, **P<0.01. Dotted lines outline the epithelium of the incisor tooth germ. Scale bar, 25 μm (**a**,**c**,**f**,**h**,**j**,**l**,**n**,**p**); 8 μm (**b**,**d**,**g**,**i**,**k**,**m**,**o**,**q**); 100 μm (**s**,**t**).

Our observation that loss of αE-catenin leads to increased nuclear YAP is consistent with earlier findings^17^. While this could be in part due to loss of the interaction between YAP and αE-catenin through 14-3-3 binding^17^, deletion of αE-catenin may also result in perturbed cell polarity, which has been recently shown to play a role in regulating YAP distribution^30^,^31^,^32^. To address this possibility, we first examined whether cell polarity is affected in the *Ctnna1* mutants by assessing the expression pattern of PAR3 and Scribble, which are markers of apical–basal and basal–lateral polarity, respectively. In the control tooth germ at E13.5, the basal epithelial layer is distinctly polarized, as indicated by the presence of PAR3 on the apical side (Fig. 2a,b) and Scribble on the lateral surface (Fig. 2f,g). In contrast, in the *Ctnna1* mutants, PAR3 was no longer apically restricted, and Scribble expression was largely downregulated (Fig. 2c–i). These results indicate that cell polarity was disrupted in the absence of αE-catenin and point to the possibility that mislocalization of YAP in *Ctnna1* mutants could be attributable to the loss of cell polarity. To test this hypothesis, we utilized the tooth explant culture system, which is a classical method for maintaining developing tooth germs *in vitro*^33^ and allows functional studies using chemical inhibitors. We disrupted the PAR/aPKC pathway that is essential for the formation and maintenance of cell polarity^34^ by inhibiting aPKC with the addition of Gö 6983 (ref. 35). This led to increased nuclear YAP in the presumptive EK region after 3 days of culturing (Fig. 2l,m,p–r), in contrast to the control tooth germs cultured with dimethyl sulfoxide (DMSO) vehicle, where low nuclear YAP was observed in the EK (Fig. 2j,k,n,o,r). Inhibition of *Par3* expression with shRNA virus in primary dental epithelial cells also increased YAP nuclear localization *in vitro* (Fig. 2s–u). These results are consistent with the notion that cell polarity is critical for YAP regulation and provide further information regarding control of YAP localization by αE-catenin.

### αE-catenin is required for EK formation

As nuclear YAP is associated with cell proliferation, we next studied whether this increase in nuclear YAP in the posterior region of the tooth germ epithelium leads to ectopic cell proliferation in *Ctnna1*^*cKO*^ by performing Ki67 staining. We examined E13.5 tooth germs, in which the early stages of the EK can be identified as a Ki67 negative non-proliferating zone at the posterior region of the control tooth germ epithelium (Fig. 1b,c). However, in the E13.5 *Ctnna1*^*cKO*^, ectopic cell proliferation was found in this region (Fig. 1d,e,u), which suggested that the EK was not forming. To further assess EK formation, we examined the expression of p21. At E13.5, when the EK is first formed, robust p21 immunostaining was observed in the control EK (Fig. 1f). In contrast, no p21-positive cells were observed in the *Ctnna1*^*cKO*^ incisor epithelium (Fig. 1g,w). Consistent with this result, *in situ* hybridization of three additional EK markers, *Shh*, *Bmp4* and *Fgf3*, confirmed the absence of the EK (Fig. 1g,j).

These results suggested that YAP might function downstream of αE-catenin to induce cell proliferation and inhibit EK formation. To test this hypothesis, we took advantage of a constitutively active *Yap* allele (*Yap*^*S127A*^)^17^, which is under the control of a tetracycline promoter. When crossed to a Cre allele in conjunction with a Cre-responsive tetracycline activator (ROSA26-rtTA)^36^, conditional expression of *Yap*^*S127A*^ can be achieved by administering tetracycline to the animal. When we drove expression of *Yap*^*S127A*^ in the incisor epithelium using *K14*^*Cre*^, a high level of nuclear YAP was detected in about 80% of the cells in the epithelium (Supplementary Fig. 2a,b). The incomplete activation of nuclear YAP is likely the result of phosphorylation of the remaining serine sites in YAP^S127A^, rendering the protein still liable for degradation. Nonetheless, by means of Student's *t*-test, we observed a significant downregulation of p21 expression and an increase in Ki67 expression in the EK region (Supplementary Fig. 2c–j). These results indicate that expression of active YAP phenocopies some aspects of the *Ctnna1*^*cKO*^ incisor phenotype and demonstrate that nuclear YAP increases cell proliferation and inhibits EK formation. However, the residual EK was sufficient to enable further development of the *Yap*^*S127A*^ tooth germ, such that incisor development was relatively normal at later stages, although the protrusion of cells on the lingual aspect seemed to be delayed (Supplementary Fig. 3).

**Figure 3 f3:**
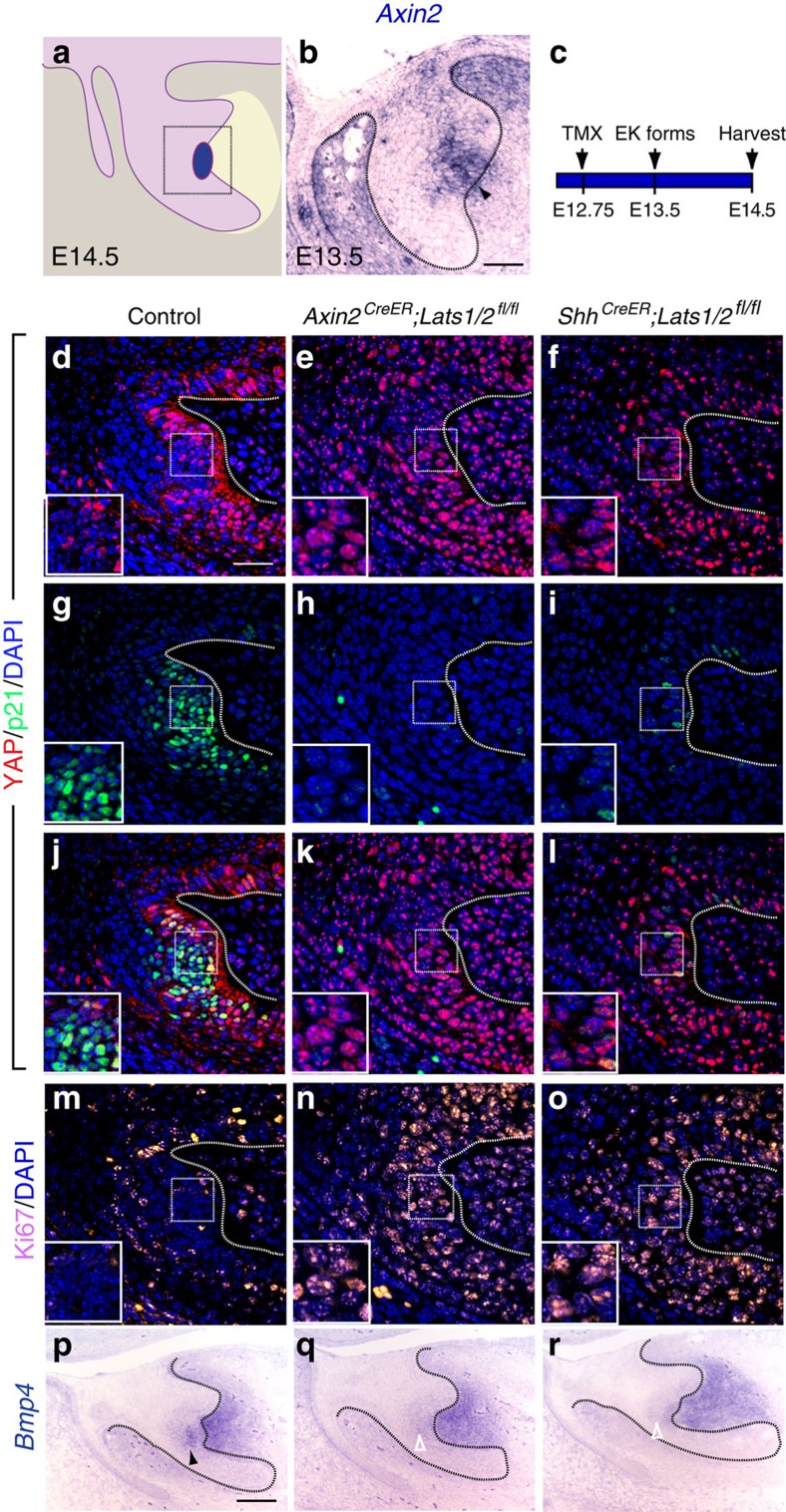
Proper YAP localization is essential for the formation of EK during tooth development. (**a**) Schematic diagram of an E14.5 incisor tooth germ. Square box represents areas imaged in **d**–**o**. (**b**) *In situ* hybridization of *Axin2* at E13.5 tooth germ (arrowhead indicates the expression of *Axin2* in the EK). (**c**) Time course for treatment of *Axin2*^*CreER*^*;Lats1/2*^*fl/fl*^ and *Shh*^*CreER*^*;Lats1/2*^*fl/fl*^ embryos with tamoxifen to delete *Lats1* and *Lats2* in the EK. (**d**,**g**,**j**,**m**) In the EK, YAP is excluded from the nucleus with increased p21 and decreased Ki67 expression at E14.5. (**e**,**f**) Double deletion of *Lats1* and *Lats2* with EK-specific *Axin2*^*CreER*^ and *Shh*^*CreER*^ induces the nuclear localization of YAP. (**h**,**i**) Double deletion of *Lats1* and *Lats2* with EK-specific *Axin2*^*CreER*^ and *Shh*^*CreER*^ decreases p21 expression. (**k**,**l**) Overlay images of YAP and p21 staining. (**n**,**o**) Double deletion of *Lats1* and *Lats2* in the EK induces ectopic cell proliferation, as shown by Ki67 staining. (**p**) *In situ* hybridization of the EK marker *Bmp4* at E14.5 (black arrowhead). (**q**,**r**) Double deletion of *Lats1* and *Lats2* inhibits *Bmp4* expression (open white arrowheads). Dotted lines outline the tooth germ epithelium in **b**–**r**. Scale bar, 50 μm (**b**,**c**); 25 μm (**d**–**o**); 100 μm **p**–**r**).

We next tested the effect of activation of nuclear YAP specifically in the EK region. To do this, we utilized *Axin2*^*CreER*^, as *Axin2* is highly expressed in the EK at E13.5 (Fig. 3b), to generate *Axin2*^*CreER*^;*Lats1*^*fl/fl*^;*Lats2*^*fl/fl*^ (*Lats1/2*^*cKO*^) embryos, where Cre recombination of *Lats1/2* is induced in the EK by delivery of tamoxifen at E12.75 (Fig. 3c). This strategy allowed better temporal control compared with the *Yap*^*S127A*^ allele, which requires administration of both tamoxifen and doxycycline for activation. In addition, as LATS1/2 phosphorylate most of the serine phosphorylation sites on YAP including S127, deletion of *Lats1/2* should result in more robust nuclear YAP localization than *Yap*^*S127A*^ misexpression in the EK, due to increased YAP stabilization. Indeed, we observed elevated nuclear YAP in the *Lats1/2*^*cKO*^ EK region at E14.5, 42 h after tamoxifen injection (compare Fig. 3e,k with Fig. 3d,j). In response to this, p21 expression was downregulated and Ki67 became apparent in the presumptive EK region (compare Fig. 3h,k,n with Fig. 3g,j,m), consistent with the *Ctnna1*^*cKO*^ phenotype (Fig. 1d,e,g). Morphologically, *Lats1/2*^*cKO*^ also lost the characteristic bulge of the EK (Fig. 3j,k). To definitively confirm loss of the EK at E14.5, we performed *Bmp4 in situ* hybridization. Both *Shh* and *Fgf3* are not adequate markers at this stage, as *Shh* expression is no longer restricted to the EK, while *Fgf3* expression is much reduced. In agreement with the p21 and Ki67 expression, the level of *Bmp4* expression was decreased in the *Lats1/2*^*cKO*^ EK region (Fig. 3p,q). A similar result was obtained when using *Shh*^*CreER*^ (Fig. 3f,i,l,o,r), which was also highly expressed in the EK region (Fig. 1h). Interestingly, the development of the tooth germ was largely normal in both cases, which is likely because both *Axin2*^*CreER*^ and *Shh*^*CreER*^ only become active during or right after the EK has formed, resulting in a relatively normal EK at E13.5 (Supplementary Fig. 4), which was not maintained at E14.5 following *Lats1/2* deletion and YAP accumulation in the nucleus.

### αE-catenin is required for incisor morphogenesis

**Figure 4:**
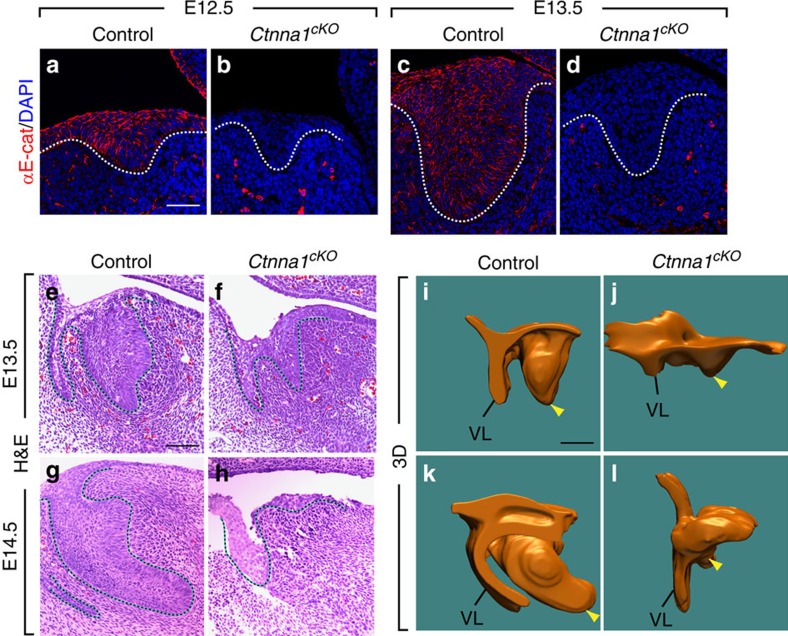
αE-catenin is required for incisor epithelium invagination. (**a**–**d**) αE-catenin is normally expressed throughout the dental epithelium at E12.5 and E13.5, but expression is absent in *Ctnna1*^*cKO*^. (**e**–**l**) Histological analysis and 3D reconstruction show smaller and abnormally shaped tooth germs at E13.5 and developmentally arrested epithelia at E14.5 in *Ctnna1*^*cKO*^ (**f**,**h**,**j**,**l**) when compared with littermate controls (**e**,**g**,**i**,**k**). Dotted lines outline the tooth germ epithelium in **a**–**h**, and yellow arrowheads indicate the tooth germ epithelium in **i**–**l**. VL, vestibular lamina. Scale bar, 25 μm (**a**–**d**); 100 μm (**e**–**h**); 200 μm (**i**–**l**).

To further investigate how deletion of *Ctnna1* and the subsequent loss of EK affect tooth development, we next characterized phenotypes associated with the epithelium itself by means of hematoxylin and eosin (H&E) staining. Such histological analysis showed that although no discernible differences were observed at E12.5 (Supplementary Fig. 5g,h), the dental epithelium was smaller in all mutants at E13.5 (Fig. 4e,f), when a range of abnormal phenotypes was observed. These phenotypes fell into three groups: a bud that lacks distinct lingual and labial tips (39%); a bud with a narrow body that tapers into a sharpened labial end but no obvious lingual protrusion (25%); or a bud with a narrow body and a rounded labial end (36%) (Supplementary Fig. 5a–d). By E14.5, growth of the tooth germ was arrested and further invagination did not occur (Fig. 4g,h). By E16.5, the incisor was absent in all αE-catenin mutants (Supplementary Fig. 5e,f). Three-dimensional (3D) reconstruction of the tooth germ epithelium provided a more comprehensive view of these defects. While the control epithelium at the posterior side began to protrude when turning posteriorly at E13.5 (Fig. 4i), the mutant dental epithelium appeared smaller and no posterior turning was observed (Fig. 4j). By E14.5, only a small epithelial remnant could be seen in *Ctnna1*^*cKO*^ (Fig. 4l), contrasting markedly with the control epithelium, where epithelial invagination continued and the anterior–posterior asymmetry became even more pronounced (Fig. 4k). This reduction in the size of the incisor epithelium was not caused by changes in cell death, as detection of apoptosis using TUNEL staining showed no difference between control and *Ctnna1*^*cKO*^ (Supplementary Fig. 5q,r).

In addition to the invagination phenotype, we also observed that cells were often lost on the oral surface and gaps could be seen within the epithelium of the *Ctnna1*^*cKO*^ (Fig. 4f,h and Supplementary Fig. 5b–d), suggesting reduced cell–cell contact consistent with the role of αE-catenin as a key component of the adherens junction. To test whether adherens junctions were affected in the absence of αE-catenin, we performed immunofluorescence staining to analyse the expression of other components of the adherens junction, E-cadherin and β-catenin. At E13.5, β-catenin was expressed throughout the tooth germ epithelium, whereas E-cadherin exhibited stronger expression in a region anterior to the EK, reminiscent of E-cadherin expression in the molar, which is also absent in the EK^37^. After deletion of *Ctnna1*, although the expression of β-catenin was unchanged, E-cadherin was upregulated in the presumptive EK region (Supplementary Fig. 5i–l). We next examined actin organization, as αE-catenin binds the actin filaments to the adherens junction. This was achieved by crossing the *K14*^*GFP-actin*^ allele^38^, which encodes a GFP–actin fusion protein, into the *Ctnna1*^*cKO*^. Whereas actin was organized properly and constriction was visible at the apical side of the control basal cells (Supplementary Fig. 5m,n), this was lost in the *Ctnna1*^*cKO*^ (Supplementary Fig. 5o,p), potentially underlying the adhesion phenotype we observed. Importantly, the reduced adhesion seen in *Ctnna1*^*cKO*^ is unlikely to be the main cause of the EK phenotype we have observed, as similar disruption in cell–cell adhesion in *K14*^*Cre*^;*Cdh1*^*fl/+*^;*Ctnnd1*^*fl/fl*^, where one copy of *Cdh1* (encoding E-cadherin) and both copies of *Ctnnd1* (encoding p120-catenin) were deleted in the dental epithelium, did not affect EK formation, as evidenced by the robust p21 expression in these mutants (Supplementary Fig. 6). Altogether, these data demonstrate that deletion of *Ctnna1* in the tooth germ epithelium leads to severe defects in incisor morphogenesis, and its role in EK formation is independent of its involvement in cell–cell adhesion.

### αE-catenin is required for mesenchymal condensation

**Figure 5:**
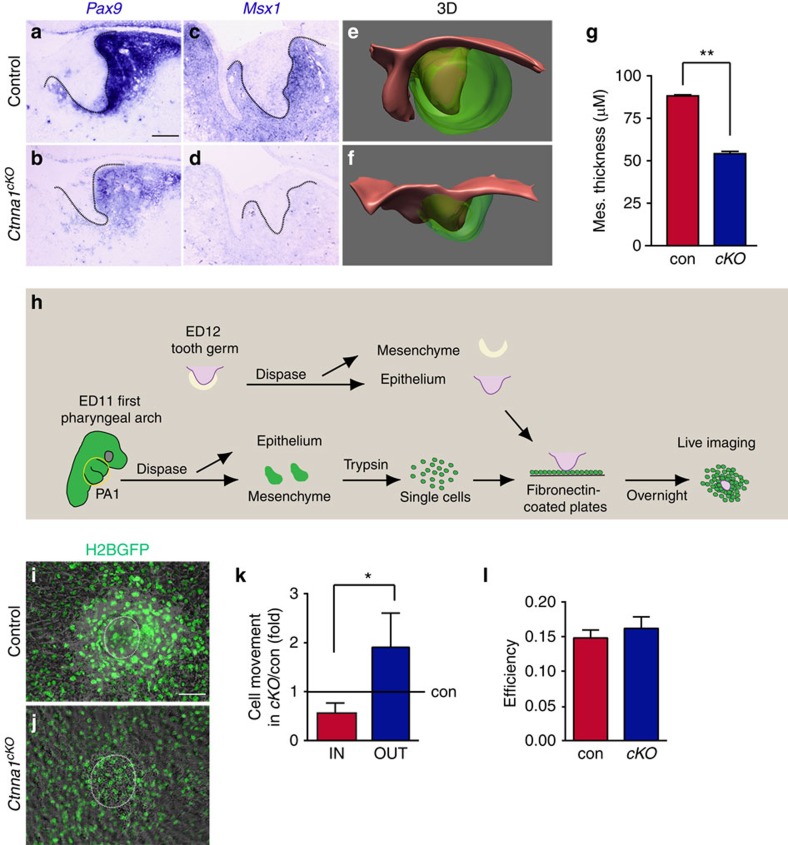
αE-catenin is required for the epithelium-induced mesenchymal condensation during incisor development. (**a**–**d**) *In situ* hybridization for *Pax9* and *Msx1* in control and *Ctnna1*^*cKO*^. In control embryos, *Pax9* and *Msx1* are expressed in the mesenchyme of the tooth germ at E13.5. In *Ctnna1*^*cKO*^, the expression of these genes is reduced or absent. (**e**,**f**) 3D reconstruction of control and *Ctnna1*^*cKO*^ tooth germs at E13.5 (epithelium is red and mesenchyme is green). The thickness of the mesenchyme is reduced in the absence of αE-catenin. (**g**) Quantification of thickness of the condensed mesenchyme around the incisor dental epithelium at E13.5 (mean±s.e.m., *n*=3, *P*<0.01). (**h**) Schematic illustration of the *in vitro* condensation assay. ED, embryonic day; PA, pharyngeal arch. (**i**,**j**) H2B-GFP labelled wild-type mesenchymal cells robustly condense around the dissected control epithelium *in vitro* (**i**) but fail to condense around the *Ctnna1*^*cKO*^ epithelium (**j**). White circles mark the position of GFP-negative epithelium. (**k**) Quantification of GFP-positive mesenchymal cells moving towards (IN) or away from (OUT) the epithelium shows *Ctnna1*^*cKO*^ epithelium is unable to retain mesenchymal cells in a condensed state (mean±s.e.m., *n*=4 in control, *n*=3 in *Ctnna1*^*cKO*^, *P*<0.05). (**l**) The efficiency of mesenchymal cell movement (displacement over distance travelled) is not affected by αE-catenin deletion (mean±s.e.m., *n*=4 in control, *n*=3 in *Ctnna1*^*cKO*^, *P*>0.05). All quantifications are analysed by using Student's *t*-test. *P<0.05, **P<0.01. Dotted lines outline the epithelium of the incisor tooth germ. Scale bar, 100 μm (**a**–**d**,**i**,**j**).

It is thought that the EK functions as a signalling centre that provides both survival and instructive signals to the underlying mesenchyme, which in turn signals back to the epithelium for proper morphogenesis^7^. As the EK failed to form in the *Ctnna1*^*cKO*^ epithelium, we next set out to determine the effects of this failure on the dental mesenchyme. To that end, we performed *in situ* hybridization to examine the expression of *Pax9*, *Msx1*, *Bmp4* and *Fgf3*, which mark the dental mesenchyme. We found that at E13.5, as previously reported^24^,^26^,^39^,^40^, *Bmp4* and *Fgf3* are expressed in both the mesenchyme and the EK, while *Pax9* and *Msx1* are only present in the mesenchyme (Figs 1j and 5a,c). In the absence of αE-catenin, *Pax9* and *Msx1* expression was downregulated in the mesenchyme (Fig. 5b,d), and the size of the *Bmp4*- and *Fgf3*-expressing domains was reduced in the mesenchyme (Fig. 1j).

We next utilized *Ctnna1* mutants to ask about roles of the EK in regulating the underlying mesenchyme. As the dental epithelium invaginates, dental mesenchymal cells begin to pack tightly around the growing epithelium^41^. Because the expression of *Pax9* and *Msx1* reflects this process^42^,^43^,^44^, their downregulation potentially indicates perturbed mesenchymal condensation^45^. We therefore compared the thickness of the condensed mesenchyme between control and *Ctnna1*^*cKO*^ tooth germs (Supplementary Fig. 7) at E13.5, which showed that the mutant mesenchyme was 1.6 times thinner than the control mesenchyme (Fig. 5e-g). To confirm that this effect in the mesenchyme was due to defects in the epithelium, we performed an *in vitro* assay based on previous reports showing that isolated molar dental epithelium can induce mesenchymal cells from the first pharyngeal arch to condense^43^. Using this approach, control or *Ctnna1*^*cKO*^ epithelium was grafted onto dissociated control mesenchymal cells that were collected from embryos expressing Histone 2B-GFP (CAG-H2B-GFP), which allowed direct visualization and tracking of each mesenchymal cell using time lapse microscopy (Fig. 5h). Whereas control epithelium was able to induce robust mesenchymal condensation, GFP (green fluorescent protein)-positive cells remained scattered in the presence of *Ctnna1*^*cKO*^ incisor epithelium (Fig. 5i,j). By tracking individual cells, we found that more cells moved away from than migrated towards the mutant epithelium compared to the control (Fig. 5k and Supplementary Movies 1–4). Thus, the *Ctnna1*^*cKO*^ epithelium did not attract mesenchymal cells as well as control epithelium, most likely as a result of the absence of the EK, which secrets a range of signalling molecules. Interestingly, the efficiency of cell movement, as defined by total displacement over total distance travelled, was unaffected (Fig. 5l), suggesting that the ability of mesenchymal cells to move is intrinsic and that signals from the EK provide directional cues to maintain continued mesenchymal condensation.

### Deletion of *Yap*/*Taz* in *Ctnna1* mutants restores EK formation

The above findings led us to hypothesize that a principal function of αE-catenin during incisor morphogenesis is to inhibit the transcriptional activity of YAP to suppress cell proliferation and promote EK formation and maintenance. As expression of constitutively active YAP did not fully repress p21 expression and EK formation, we suspected that additional factors, such as the YAP homologue TAZ, may be required to regulate EK formation. Similar to YAP, TAZ localization can be regulated by αE-catenin^46^. We therefore set out to test the hypothesis that combined deletion of *Yap* and *Taz* in the *Ctnna1*^*cKO*^ mutants would rescue the αE-catenin mutant phenotype.

**Figure 6:**
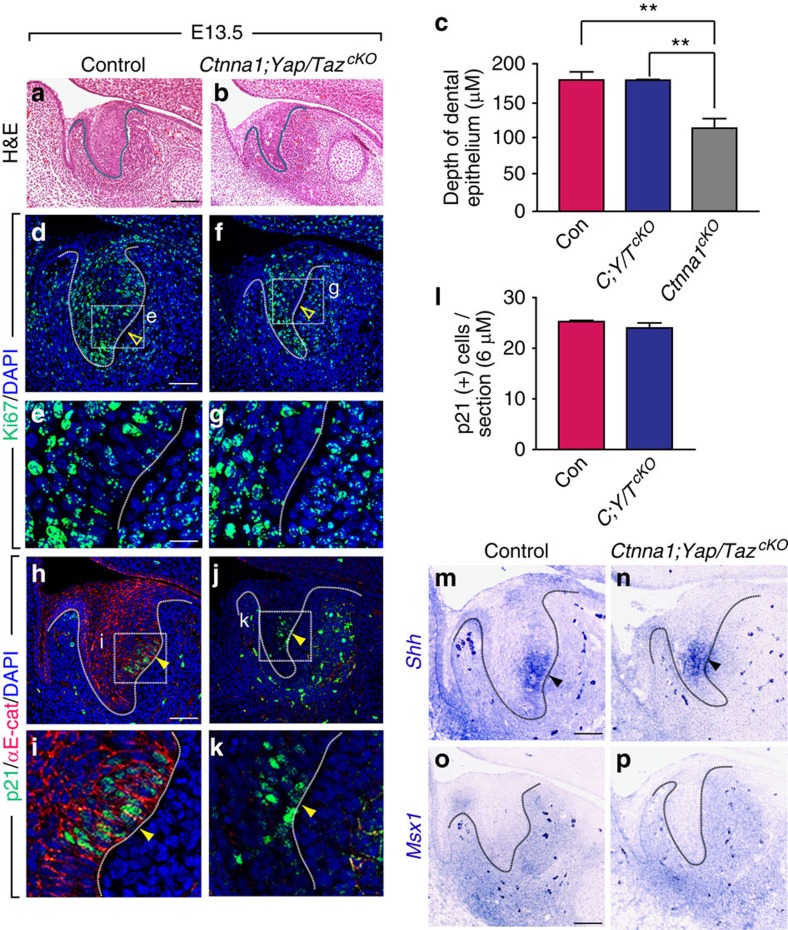
αE-catenin regulates enamel knot formation and tooth germ invagination by controlling YAP/TAZ activity during tooth development. (**a**,**b**) H&E staining of E13.5 tooth germ shows that double deletion of *Yap*/*Taz* in the *Ctnna1*^*cKO*^ dental epithelium is able to rescue the invagination defect seen in *Ctnna1*^*cKO*^. (**c**) Quantification of the depth of invaginated tooth germ in control, triple knockouts and *Ctnna1*^*cKO*^ at E13.5 (mean±s.e.m., *n*=3, *P*<0.01). (**d**–**g**) Deletion of *Yap*/*Taz* in *Ctnna1*^*cKO*^ restores the non-proliferating zone at the posterior region of the tooth germ, as assessed by Ki67 staining, indicating the formation of the EK (open yellow arrowheads). (**h**–**k**) p21 staining confirms that deletion of *Yap*/*Taz* in *Ctnna1*^*cKO*^ reestablishes a non-proliferating zone, indicating the formation of the EK in the tooth germ (yellow arrowheads). (**l**) Quantification of the number of p21-positive cells per section in control and triple knockout tooth germs (mean±s.e.m., *n*=3, *P*>0.05). (**m**,**n**) *Shh* expression at the posterior region of the tooth germ epithelium is rescued on *Yap/Taz* deletion in the *Ctnna1*^*cKO*^ (dark arrowheads). (**o**,**p**) Expression of the mesenchymal gene, *Msx1,* is rescued in the dental mesenchyme at E13.5 after deletion of *Yap*/*Taz* in *Ctnna1*^*cKO*^ in the tooth germ. All quantifications are analysed by using Student's *t*-test. *P<0.05, **P<0.01. Dotted lines outline the epithelium of the incisor tooth germ. Scale bar, 100 μm (**a**,**b**); 50 μm (**d**,**f**,**h**,**j**,**m**–**p**); 8 μm (**e**,**g**,**i**,**k**).

To that end, we generated *K14*^*Cre*^;*Ctnna1*^*cKO*^;*Yap*^*cKO*^;*Taz*^*cKO*^ triple mutants to analyse tooth germ morphology, cell proliferation and EK formation. Immunostaining of YAP showed effective removal of the protein in these mutants (Supplementary Fig. 8a–d). Interestingly, deletion of just *Yap* and *Taz* using *K14*^*Cre*^ did not have a marked effect on the morphology of the developing incisor, and the EK was fully formed (Supplementary Fig. 9), indicating that compensatory mechanisms can support cellular proliferation in the absence of *Yap* and *Taz*. Remarkably, however, the *K14*^*Cre*^;*Ctnna1*^*cKO*^;*Yap*^*cKO*^;*Taz*^*cKO*^ triple mutants exhibited a tooth germ that had a comparable depth of invagination as the control at E13.5, although the width of the mutants remained decreased (Fig. 6a–c). Importantly, the Ki67-negative region in the triple mutants was re-established at the posterior side of the basal layer (Fig. 6d–g and compare with Fig. 1d,e), and p21-positive cells were again detected in this region (Fig. 6h–l and compare Fig. 6j,k with Fig. 1g). *In situ* hybridization showed that *Shh* was expressed in the presumptive EK region (Fig. 6m,n), which demonstrated that combined deletion of *Yap* and *Taz* rescued not only the cell biological features but also the molecular signals in the *Ctnna1*^*cKO*^ EK.

**Figure 7:**
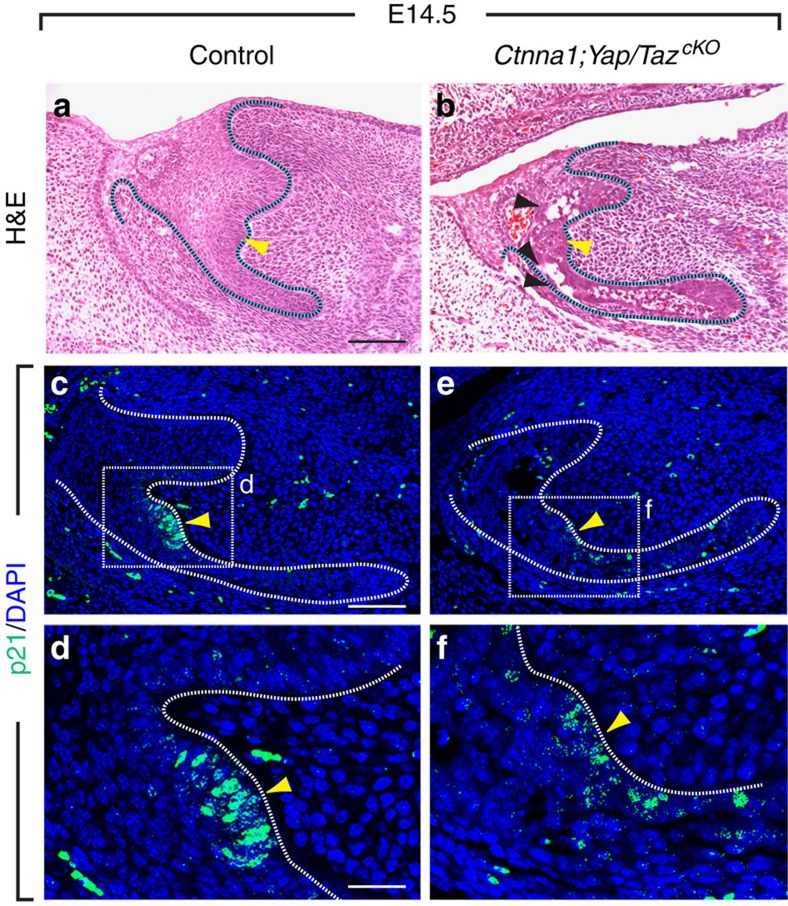
The αE-catenin-YAP/TAZ signalling axis is required for further tooth germ invagination and development. (**a**,**b**) H&E staining of E14.5 tooth germ shows that the *Ctnna1*^*cKO*^ tooth germs passes the bud stage and progress to the cap stage after deletion of *Yap*/*Taz*. Black arrowheads mark tears within the tissue and yellow arrowheads mark EK. (**c**–**f**) p21-expressing cells (yellow arrowheads) are maintained at E14.5 in the *Ctnna1*^*cKO*^ tooth germ after deletion of *Yap*/*Taz*. Scale bar, 100 μm (**a**,**b**,**c**,**e**); 25 μm (**d**,**f**).

Next, we examined whether restoration of the EK in the triple mutants rescued the mesenchymal defect in *Ctnna1*^*cKO*^. *In situ* hybridization showed that *Msx1* expression was recovered in the mesenchyme to a level that was comparable to that in control (Fig. 6o,p), suggesting that the epithelial–mesenchymal feedback loop was re-established. Strikingly, histological analysis at E14.5 showed that the tooth germ in the triple mutants invaginated further and developed to cap stage (Fig. 7a,b). This was in stark contrast to the *Ctnna1*^*cKO*^, where dental epithelium could barely be detected at this stage (compare Fig. 7b with Fig. 4h). p21 staining also showed that the EK was maintained at E14.5 after deletion of *Yap*/*Taz* in the *Ctnna1*^*cKO*^ (Fig. 7c–f). Interestingly, disruptions were still apparent in sections of the triple mutants (Fig. 7b), providing further evidence that adhesive functions of αE-catenin during tooth development are separable from its role in EK formation, which involves the regulation of YAP/TAZ signalling. In this context, YAP appeared to play a more prominent role than TAZ, as *Ctnna1*^*cKO*^;*Yap*^*cKO*^;*Taz*^*het*^ tooth germs continued to invaginate at E16.5 (Supplementary Fig. 8g,h) and *Ctnna1*^*cKO*^;*Yap*^*het*^;*Taz*^*cKO*^ failed to do so (Supplementary Fig. 8e,f).

## Discussion

A central event during tooth development is the formation of the EK, during which a group of proliferating cells exit the cell cycle, become densely packed at the posterior side of the epithelium, and begin to express an array of signalling molecules. However, the processes that regulate EK formation remain a mystery. Our observation that EK cells are devoid of nuclear YAP suggested that an active mechanism may be in place to regulate its localization and thus controls cell proliferation and EK formation. This was demonstrated through our conditional deletion of αE-catenin in the incisor epithelium. In the absence of αE-catenin, YAP accumulated in the nucleus of cells in the presumptive EK region, which continued to proliferate and failed to express the cell cycle inhibitor p21 and other EK markers, including *Shh*, *Bmp4* and *Fgf3*. Thus, the loss of *Ctnna1* results in the complete absence of the EK. Importantly, this phenotype is due to the increased nuclear accumulation of YAP that acts cell-autonomously within the EK region, as mutants with EK-specific deletion of *Lats1/2* failed to maintain the EK. This change in YAP activity accounts for the ectopic proliferation we observed, as expression of a constitutively active YAP (*Yap*^*S127A*^) or deletion of *Lats1*/*2*-induced YAP nuclear localization and was sufficient to drive proliferation in the EK as well as downregulate p21 expression. It is therefore paradoxical at first glance that *Ctnna1* mutant tooth germs are smaller than the control, despite increased proliferation in the EK. However, as the EK is a relatively small structure within the developing tooth, and cell proliferation is unchanged in the rest of the tissue, the increase in proliferation within the EK is not sufficient to compensate for loss of EK function in terms of the contribution to overall size. It should also be noted that the extent of inhibition of nuclear YAP by αE-catenin is different between EK and non-EK epithelial cells, suggesting that additional mechanisms must exist to either further restrict YAP in the cytoplasm in the EK or promote nuclear YAP accumulation in the non-EK cells. Interestingly, YAP and TAZ are dispensable for tooth germ invagination up to the bell stage (Supplementary Fig. 9)^47^, indicating that additional mechanism(s) may be in place to compensate for their functions, and further studies are required to examine the role of YAP/TAZ in the non-EK epithelium.

Our finding that nuclear YAP affects EK formation in the incisor is concordant with a recent paper that also utilized *Yap*^*S127A*^ to study YAP function in the developing molar. In that paper, *Shh* expression in the presumptive EK region was similarly inhibited by the constitutively active YAP. Interestingly, an ectopic EK was reported in the molar in this paper; however, this finding is highly variable and occurs only on certain genetic backgrounds (X.-P. Wang, personal communication). We examined *K14*^*Cre*^*;Yap*^*S127A*^ molars in our embryos and found that, in contrast to the earlier report, EK formation was inhibited without the induction of an ectopic EK (Supplementary Fig. 2k–t). Given the dependence on genetic background, the difference between our findings and the previous report are likely caused by the use of mice with different genetic backgrounds.

To our knowledge, the *Ctnna1*^*cKO*^ is the first mutation shown to affect all aspects of EK formation, including cessation of cell proliferation and the production of signalling molecules. For example, in the *downless* (*Edar*) or *tabby* (*Eda*) mutant molars, although the EK forms and expresses p21 as well as *Shh*, *Fgf4* and *Bmp4*, the organization and the shape of the EK are dramatically altered^48^. In *Lef1*^*−/−*^ embryos, the EK is formed, as evidenced by the expression of p21, but key signalling molecules, such as *Shh*, *Fgf3* and *Fgf4*, are not expressed^49^. Therefore, αE-catenin may function upstream of these genes to first establish a group of non-proliferating cells, which are further regulated by EDA/EDAR and LEF1. Signals from the condensing dental mesenchyme such as BMP4 are also important for the establishment of the EK. By means of bead implantation, BMP4 was shown to be capable of inducing p21 expression^5^, and in *Msx1* null mice, where *Bmp4* expression is abrogated, cells in the presumptive EK region continue to proliferate, exhibiting a cell division phenotype similar to *Ctnna1*^*cKO*^ (ref. 50). Thus, αE-catenin may function within the epithelium in conjunction with BMP4 to regulate p21 expression and the cell cycle during formation of the EK.

The disappearance of the EK in αE-catenin mutants presented an opportunity to test the functional requirement of the EK during odontogenesis. Since the discovery many years ago that the EK expresses an array of signalling molecules, it has been widely held that this structure acts as a signalling centre, guiding the development of the tooth. This notion is supported by several classical bead implantation experiments demonstrating that molecules secreted from the EK, such as FGFs, are sufficient to induce cell proliferation in both the non-EK epithelium and the mesenchyme, providing a mechanism for regulation of epithelial folding and the transition from the bud to the cap stage^8^,^51^. Similar experimental approaches have also been used to show that EK factors can induce expression of mesenchymal genes such as *Runx2* (ref. 52). However, these gain-of-function studies did not explicitly test the functional requirement of the EK. Experiments using global gene inactivation, where the gene of interest is deleted in the entire animal, also could not determine whether the phenotypes were caused by the lack of the EK or by altered mesenchymal development. Here we show that absence of the EK as a result of epithelial deletion of *Ctnna1* leads to failure of the dental mesenchyme to condense and express markers associated with condensation, such as *Pax9* and *Msx1*, as well as signalling molecules, such as *Bmp4* and *Fgf3*, which are important for odontogenesis and the regulation of the reciprocal epithelial–mesenchymal signalling pathways. This is similar to the *Lef1* null mice, where LEF1 is required to induce *Fgf3* expression in the mesenchyme through activation of epithelial FGF4 in molars. FGF3 in turn signals the epithelium to further regulate its development^49^. In *Fgfr2(IIIb)*^−/−^ embryos, the molar tooth germs do not develop normally and fail to progress beyond the cap stage due to the decreased dental epithelial cell proliferation and loss of *Fgf3*/*4* and *Bmp4* expression in the EK^53^,^54^. Altogether, these results, along with our findings, show that the interaction between the EK and the mesenchyme by means of FGF and BMP signalling are important in maintaining mesenchymal condensation and proper epithelial cell proliferation and invagination. However, there remains the possibility that the mesenchymal phenotypes we have observed were a result of defects in the *Ctnna1* non-EK epithelium, as currently there are no tools available to specifically disrupt the EK function before its formation.

Our finding that YAP functions downstream of αE-catenin mirrors what has been observed in the skin, where expression of *Yap*^*S127A*^ causes hyperproliferation, and deletion of *Ctnna1* leads to increased nuclear YAP^17^. However, in contrast to observations made in the skin or during liver repair^12^,^17^, we did not observe hyperplasia and tumour formation in the tooth germ as a result of *Ctnna1* deletion, but rather perturbed tissue morphogenesis due to failed cell cycle arrest and EK formation. These data thus suggest that the αE-catenin–YAP signalling axis is important both during embryonic development and adult tissue homeostasis, despite having distinct phenotypic outputs. However, how αE-catenin regulates YAP localization remains unclear. In the epidermis, 14-3-3 functions as an intermediate that connects αE-catenin to YAP and maintains YAP cytoplasmic localization in a LATS1/2- and MST1/2-independent manner^17^. αE-catenin further inhibits YAP activity by attenuating SRC function, which phosphorylates Y341/357/394 of YAP to promote YAP nuclear localization and transcriptional activity^55^. However, in other contexts, αE-catenin does seem to intersect with the Hippo core pathway. The LIM protein, Ajuba, can interact with αE-catenin and has been shown to interact with LATS1 and inhibit YAP phosphorylation^56^,^57^. Recently, it was also found in the epidermis that αE-catenin interacts with the neurofibromatosis type 2 (NF2) tumour suppressor, an upstream molecule in the Hippo pathway that bridges αE-catenin to PAR3 (ref. 58). Deletion of NF2 in the epidermis phenocopies αE-catenin null phenotypes. PAR3 itself can activate TAZ activity by inducing LATS1 dephosphorylation and inhibition through protein phosphatase 1 (PP1) interaction^59^. In the incisor tooth germ, we found that αE-catenin is required to maintain PAR3 apical localization and cell polarity, which in turn is critical for regulating YAP localization. This finding is consistent with previous reports in both *Drosophila* and mice that proper polarization of cells is required for correct YAP localization^30^,^31^. In addition, as deletion of *Lats1/2* in the incisor epithelium induces nuclear YAP, the αE-catenin/PAR3 axis could potentially act upstream or in parallel of LATS1/2 to regulate YAP localization in the EK. Altogether, these results establish that αE-catenin is essential for normal tissue homeostasis via restriction of YAP in the cytoplasm through at least two distinct mechanisms. We next wanted to ask whether the increase in nuclear YAP is the main cause to the phenotypes observed. To tackle this question, we directly tested whether the increase in nuclear YAP and/or TAZ is required for the manifestation of the loss of αE-catenin by deleting both *Yap* and *Taz* in the *Ctnna1* mutants. To our knowledge, this was the first attempted *in vivo* genetic rescue of a *Ctnna1*^*cKO*^ phenotype by deletion of *Yap/Taz*. Remarkably, the removal of both YAP and TAZ in the *Ctnna1* mutants not only restored the EK structure and its function but also enabled the tooth germ to continue invaginating. This leads us to conclude that during tooth development, αE-catenin facilitates the formation of the EK by restricting YAP/TAZ in the cytoplasm, permitting the cells to exit the cell cycle and become specialized for signal production.

One question that will be of great future interest is to further dissect the cell adhesion versus signalling functions of αE-catenin in formation of the EK. It is possible that during EK formation the adhesive function of αE-catenin is involved in the initial process of cell condensation, and that the protein subsequently has a second function of stopping proliferation based on regulation of YAP/TAZ. The rescue of tooth invagination in the *K14*^*Cre*^;*Ctnna1*^*fl/fl*^;*Yap*^*fl/fl*^;*Taz*^*fl/fl*^ triple mutants indicates that the cell adhesive function is not required for formation of the EK and progression to the cap stage, suggesting that other molecules could be involved in the regulation of cell condensation during EK formation. Interestingly, *K14*^*Cre*^;*Cdh1*^*fl/+*^;*Ctnnd1*^*fl/fl*^ mutant embryos exhibited cell adhesion defects in the tooth germ, but had otherwise normal EK formation and tooth development (Supplementary Fig. 6). These data support the notion that several aspects of early odontogenesis, including EK formation, epithelial invagination and mesenchymal condensation after lamina stage, are independent of cadherin mediated cell–cell adhesion, and instead rely on other mechanisms, such as correct maintenance of cell polarity and signal regulation of YAP.

Signalling centres are the chief guiding force for the patterning and formation of many organs during development. However, the molecular and cellular mechanism underlying their formation remains less understood. Many signalling centres, such as the AER, floor plate and isthmus, have much reduced cell proliferation in comparison with the neighbouring cells^11^,^60^. This observation suggests that perhaps similar mechanisms are involved in formation of these signalling centres by causing cells to exit the cell cycle before becoming specialized in signal production. During formation of the EK, we have identified αE-catenin-dependent YAP/TAZ inhibition as a critical process for cells to commence the expression of p21 and cease proliferating. Interestingly, several signalling centres we have examined (AER, floor plate and isthmus) all express αE-catenin (Supplementary Fig. 1t–x) and have reduced nuclear YAP staining, low Ki67 labelling, and high p21 expression (Supplementary Fig. 1a–r). While this is a correlative observation, we suggest that some aspects of the mechanistic regulation of EK formation exist in the development of other signalling centres, which may also require αE-catenin to actively restrain nuclear YAP/TAZ accumulation and proliferation, allowing cell differentiation of a group of signal-producing cells.

## Methods

### Mouse lines and induction of alleles

*K14-Cre* (ref. 28), *K14-actin-GFP* (ref. 38), *CAG-H2B-EGFP* (ref. 61), *Yap*^*S127A*^ (ref. 17), *Shh^CreER^* (ref. 62), *Axin2^CreER^* (ref. 63) and conditional alleles of *Ctnna1* (ref. 29), *Lats1* and *Lats2* (refs 64, 65), *Cdh1* (ref. 66), *Ctnnd1* (ref. 67), *Yap* and *Taz*^68^,^69^ were maintained and genotyped as previously reported. The mice maintained on mixed genetic backgrounds as a result of breeding between different lines. Age-matched littermates were used as controls in all cases, and both male and female embryos were included in all experiments. When *Shh^CreER^* or *Axin2^CreER^* mice were used, tamoxifen was delivered to the pregnant females through intraperitoneal injection at stages indicated within the text. Activation of *Yap*^*S127A*^ was achieved through provision of doxycycline in food starting at E9.5. All experiments involving mice were approved by the Institutional Animal Care and Use Committee of the University of California, San Francisco (protocol #AN099613).

### Tissue preparation and histological analysis

E12.5 to E16.5 embryos were fixed with 4% paraformaldehyde (PFA) in PBS overnight at 4 °C. Embryos were embedded in paraffin. Sections were cut at 6 μm and stained with H&E using standard methods.

### Immunofluorescence staining

For immunofluorescence, paraffin sections were rehydrated, and antigen retrieval was performed with citrate buffer containing 2 mM EDTA, 0.05% Tween 20 in a pressure cooker. Antibodies and dilutions used were as follows: αE-catenin (mouse, 1:500; BD Transduction Laboratories), β-catenin (rabbit, 1:1,000; Abcam), BrdU (rat, 1:500; Invitrogen), Ki67 (rat, 1:100; DAKO), GFP (rabbit, 1:1,000; Abcam), p21 (mouse, 1:500; BD Transduction Laboratories), YAP (rabbit, 1:100; Cell Signalling), and pYAP (rabbit, 1:100; Cell Signalling), E-cadherin (rat, 1:1,000; Zymed), Scribble (goat, 1:100; Cell signalling), PAR3 (rabbit, 1:500; Millipore). Secondary antibodies were coupled to Alexa 488, Alexa 555 (Invitrogen), or Biotin (Vector). TSA kit (PerkinElmer) was used for signal amplification for p21, YAP and pYAP detection. Nuclear counterstaining was performed with DAPI (Invitrogen), and all images were acquired using a Leica-TCS SP5 confocal microscope.

### *In situ* hybridization

Sections (6 μm) were prepared and RNA *in situ* hybridization was performed on tissue sections using digoxigenin-labelled probes according to standard protocols. Alkaline phosphatase activity was detected on tissue sections using BM Purple staining solution (Roche) after overnight incubation in alkaline phosphatase buffer. Brightfield images were obtained using a Leica DFC 500 camera with a Leica DM 5000B microscope.

### 3D analysis of embryonic incisors

The epithelium and mesenchyme were outlined from 30 to 60 consecutive 4-μm-thick sagittal sections (*n*=3 animals per genotype). BioVis software (http://www.biovis3d.com) was used to generate 3D reconstructions.

### Incisor explant culture

Lower mandibles were dissected out from E12.5 embryos and tongues were bladed away with fine needle in cold media. Mandibles were seeded into matrigel (20 μl) supplemented with 10 μM of Gö 6983 or DMSO control and laid into the cell insert with 350 μl of complete DMEM in the bottom. The complete DMEM was similarly supplemented with 10 μM of Gö 6983 (Selleckchem) or DMSO. Tissues were cultured for 72 h with changing of media every day. After 72 h, tissues were harvested and fixed with 4% PFA. Tissues were processed for paraffin section and the middle three sections were stained with YAP antibody to visualize its localization.

### Lentivirus infection of primary dental epithelial cells

Dental epithelial cells from E12.5 tooth germs were infected using ∼25 μl of lentiviral supernatant (control shRNA and *Par3* shRNA, Santa Cruz Biotechnology) with 5 μg ml^−1^ polybrene. Infections were typically performed in 6-well plates seeded with 15,000 cells. 12 h after infection, the supernatant was removed and cells were cultured with new media. Two days following infection, cells were fixed with 2.5% PFA and stained with YAP antibody.

### Determination of the condensed mesenchymal region

The condensed mesenchyme can be easily distinguished from the surrounding non-condensed mesenchyme by visually inspecting H&E staining and a clear border can be drawn between the two regions, similar to earlier observations^43^,^44^,^45^. To statistically verify that we had indeed demarcated the two mesenchymal regions, we measured and compared the degree of coherence (similarity in cell orientation of adjacent cells^70^) within the condensed and non-condensed mesenchyme. Briefly, cell orientation angles were determined using ImageJ, and for each cell, the difference in cell orientation was calculated for cells within 50-pixels distance and used to derive the s.d. for this subset. This was repeated for all cells and an average of the s.d. was acquired for cells within the condensed and non-condensed mesenchyme. A Student's *t-*test was then used to determine whether the coherence between these two regions was statistically distinct.

### *In vitro* condensation and live imaging

For this assay, the published method^43^ was used with slight modification. The first pharyngeal arch was dissected from CAG-H2B-EGFP E11 embryos and treated with Dispase II (2.4 U ml^−1^; Roche) at 37 °C for 13 min. After a 13-min incubation, 3 ml DMEM was added to stop digestion. The epithelium was removed and the dental mesenchyme was dissected out and incubated with 0.05% trypsin for 1 min, gently triturated several times, and 8 × 10^4^ cells plated into 12-well fibronectin (Becton Dickinson)-coated glass bottom dishes (MatTek Corporation) in 400 μl DMEM supplemented with 10% fetal bovine serum. The media was changed the next day, and after 2 days of culturing, when the cells reached around 90% confluence, freshly isolated E12 dental epithelium from controls and *Ctnna1* mutants were overlaid on top of the monolayer of GFP-positive mesenchymal cells and cultured for 48 h. For live imaging, after overnight incubation to let the epithelium adhere to the mesenchymal cells, images were acquired every 4 min for 24 h using a Leica LP5 confocal microscope.

### μCT imaging analysis

Mandibles from E16.5 embryos were harvested and dehydrated through ethanol series to 70% ethanol. Samples were then soaked in phosphotungstic acid overnight to differentially stain soft tissues for μCT visualization using MicroXCT-200 (Xradia) with a spatial resolution of 0.5 μm. Images acquired were analysed using Avizo (VSG).

### Statistical analysis

Data were reported as means±s.e.m. Unpaired Student's *t*-test was performed where significance was indicated by two-tailed *P* values; *P<0.05, **P<0.01 and ***P<0.001.

### Data availability

The authors declare that the data supporting the findings of this study are available within the article and its Supplementary Information files.

## Additional information

**How to cite this article:** Li, C.-Y. *et al.* αE-catenin inhibits YAP/TAZ activity to regulate signalling centre formation during tooth development. *Nat. Commun.* 7:12133 doi: 10.1038/ncomms12133 (2016).

## Supplementary Material

Supplementary Information Supplementary Figures 1-9

Supplementary Movie 1 Time-lapse microscopy shows H2B-GFP positive mesenchymal cells condense around the GFP negative control dental epithelium over a span of 24 hours.

Supplementary Movie 2 Automated cell tracking of Supplementary Movie 1 using Imaris.

Supplementary Movie 3 Time-lapse microscopy shows H2B-GFP positive mesenchymal cells fail to condense around the GFP negative *Ctnna1^cKO^* dental epithelium over a span of 24 hours.

Supplementary Movie 4 Automated cell tracking of Supplementary Movie 3 using Imaris.
