# Supplementary material for: αE-catenin inhibits YAP/TAZ activity to regulate signalling centre formation during tooth development
Source: Nat Commun. 2016 Jul 13;7:12133. doi: 10.1038/ncomms12133 (PMC4947177; doi:10.1038/ncomms12133)
Supplement: Supplementary Information — Supplementary Figures 1-9 [file ncomms12133-s1.pdf]

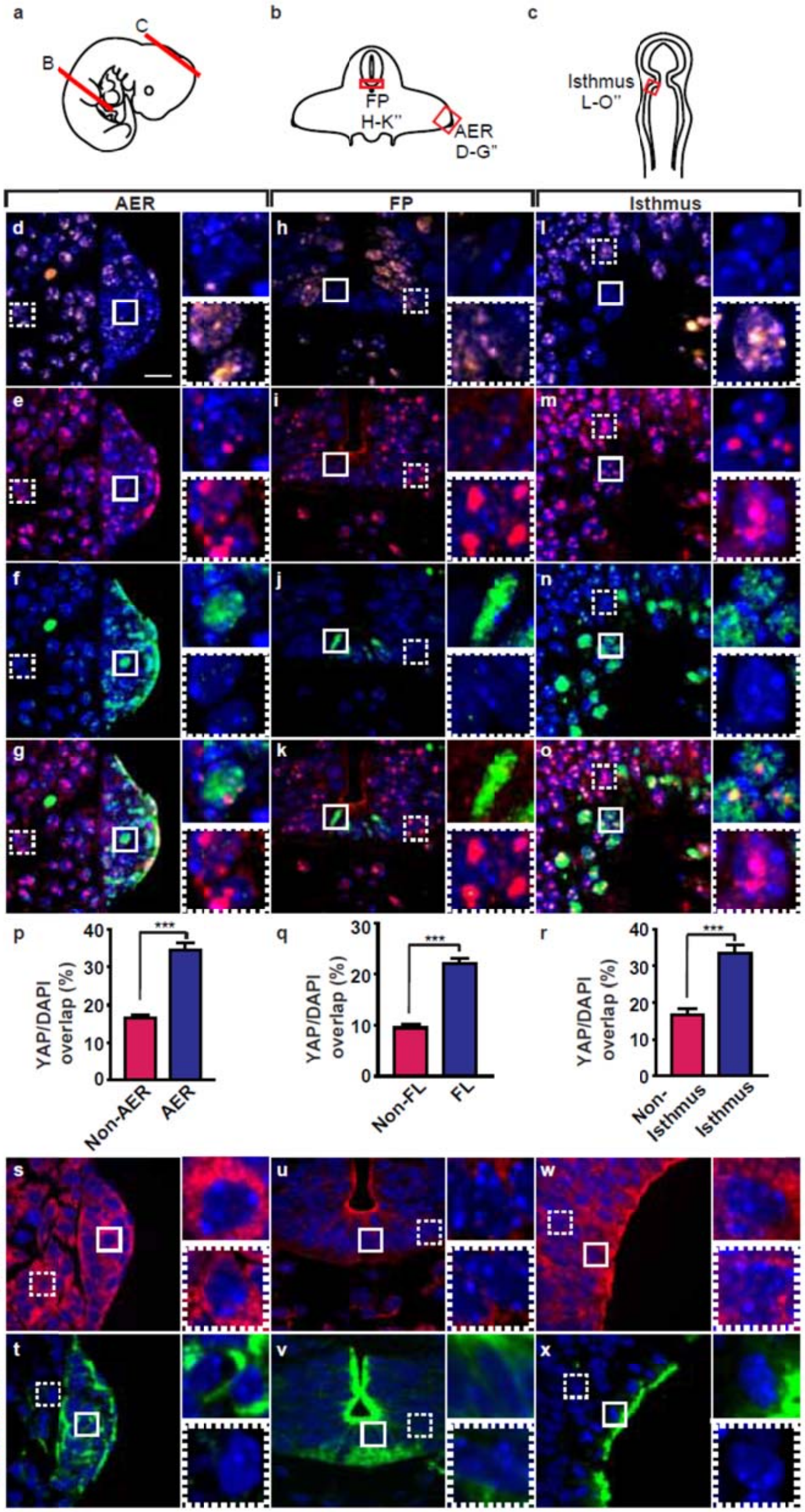

a
b
c
B
C
FP
H-K''
AER
D-G''
Isthmus
L-O''
AER
FP
Isthmus
Ki67/DAPI
YAP/DAPI
P21/DAPI
YAP/p21/DAPI
YAP/DAPI overlap (%)
Non-AER
AER
Non-FL
FL
Non-Isthmus
Isthmus
***
pYAP/DAPI
αE-cat/DAPI

Supplementary Figure 1: Signaling centers contain few proliferating cells, express p21, and exclude YAP from the nucleus. (a) Schematic diagram of an E10.5 mouse embryo. **(b,c)** Sections at B and C in (a) are generated to study the apical ectoderm ridge (AER), floor plate (FP) (b) and isthmus (c). **(d-o)** Triple staining of YAP (red), p21 (green), and Ki67 (orange) in the AER of the limb (d-g), floor plate of the neural tube (h-k), and isthmus (l-o) at E10.5 shows low Ki67 staining, high p21 expression, and the lack of nuclear YAP in each signaling center. Representative cells within the signaling centers (solid box) and the surrounding tissues (dashed box) are enlarged respectively. **(p-r)** Quantification of overlapping signals of YAP and DAPI in each signaling center (mean ± SEM, n=5, $p<0.05$). **(s,u,w)** Expression of S127 phosphorylated YAP in the signaling centers. **(t,v,x)** Expression of αE-catenin in the signaling centers. All quantifications are analyzed by using Student's t-test.Yellow arrowheads indicate the signaling center in AER, the floor plate and the isthmus. Scale bar, 20 μm in d-o,s-x.

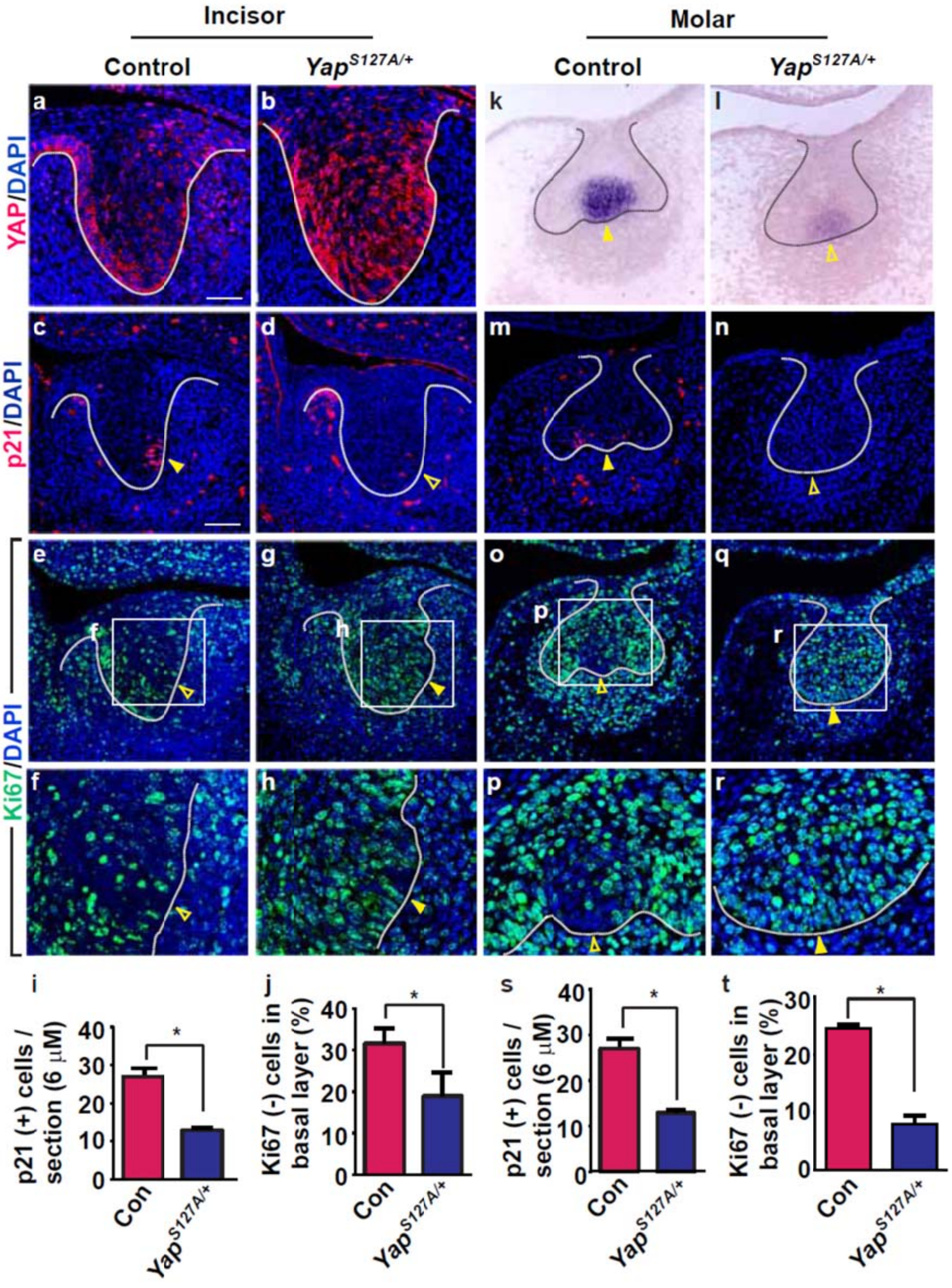

Supplementary Figure 2: Expression of constitutively active YAP induces ectopic cell proliferation and inhibits the EK formation during tooth development. (a,b) Expression of a

constitutively activated YAP (*Yap*^{S127A}) at E13.5 results in increased nuclear YAP. **(c,d)** Expression of *Yap*^{S127A} results in reduced p21 expression in the EK (open yellow arrowhead in d compared to yellow arrowhead in c). **(e-h)** Ki67 negative cells are decreased in numbers at the posterior region of the incisor tooth germ at E13.5 after *Yap*^{S127A} expression (yellow arrowheads in g,h) compared to control (open yellow arrowheads in e,f). **(i)** Quantification of the number of p21 positive cells at the posterior region of the epithelium in the incisor tooth germ (mean ± SEM, n=3, $p<0.05$). **(j)** Quantification of the number of Ki67 negative cells at the posterior region of the dental epithelium in the incisor tooth germ (mean ± SEM, n=3, $p<0.05$). **(k,l)** *Shh* expression is decreased at E14.5 after *Yap*^{S127A} expression in the molar tooth germ. **(m,n)** Expression of *Yap*^{S127A} results in reduced p21 expression in the molar EK (open yellow arrowhead in l compared to yellow arrowhead in k). **(o-r)** Ki67 negative cells are decreased in numbers at the basal region of the molar tooth germ at E14.5 after *Yap*^{S127A} expression (yellow arrowheads in q,r) compared to control (open yellow arrowheads in o,p). **(s)** Quantification of the number of p21 positive cells at the basal region of the epithelium in the molar tooth germ (mean ± SEM, n=3, $p<0.05$). **(t)** Quantification of the number of Ki67 negative cells at the basal region of the dental epithelium in the molar tooth germ (mean ± SEM, n=3, $p<0.05$). All quantifications are analyzed by using Student's t-test. Dotted lines outline the epithelium of the incisor tooth germ. Scale bars, 25 µm in a,b; 50 µm in c-e,g, m-n,o,q; 8 µm in f,h,p,r .

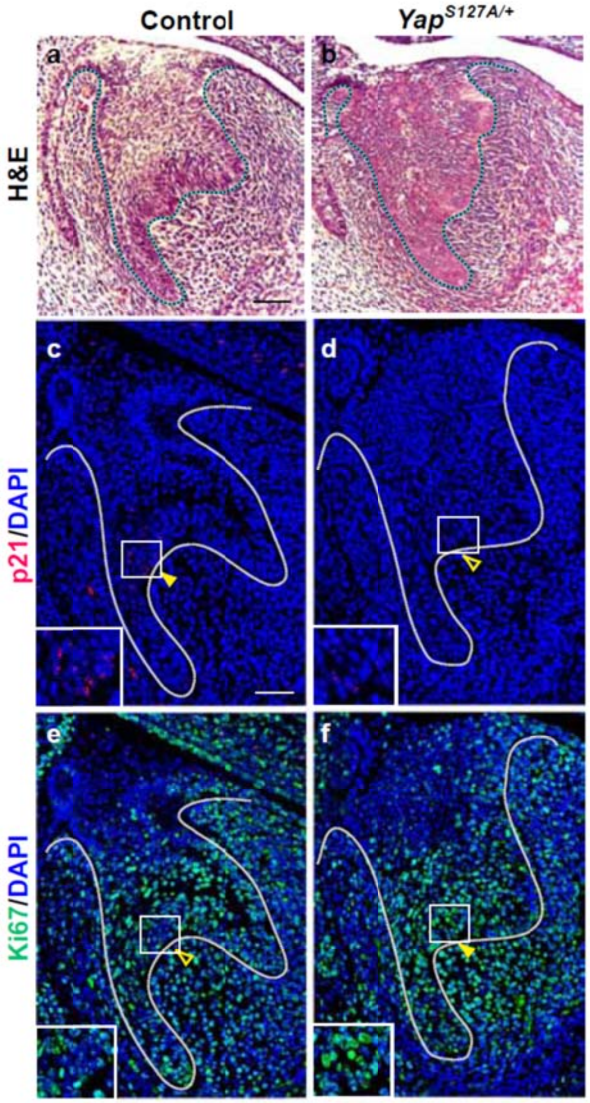

Supplementary Figure 3: Expression of a constitutively active YAP delays invagination of lingual epithelium. (a,b) Expression of a constitutively active YAP (*Yap*^{S127A}) results in delayed invagination of the lingual epithelium at E15.5. **(c,d)** Expression of *Yap*^{S127A} results in reduced p21 expression in the EK (compare open yellow arrowhead in d to yellow arrowhead in c). **(e,f)** Ki67 negative cells are decreased in numbers at the posterior region of the incisor tooth germ at E15.5 after *Yap*^{S127A} expression (yellow arrowhead in f) compared to the control (open yellow arrowhead in e). Dotted lines outline the epithelium of the incisor tooth germ. Scale bars, 75 µm in a,b; 50 µm in c-f .

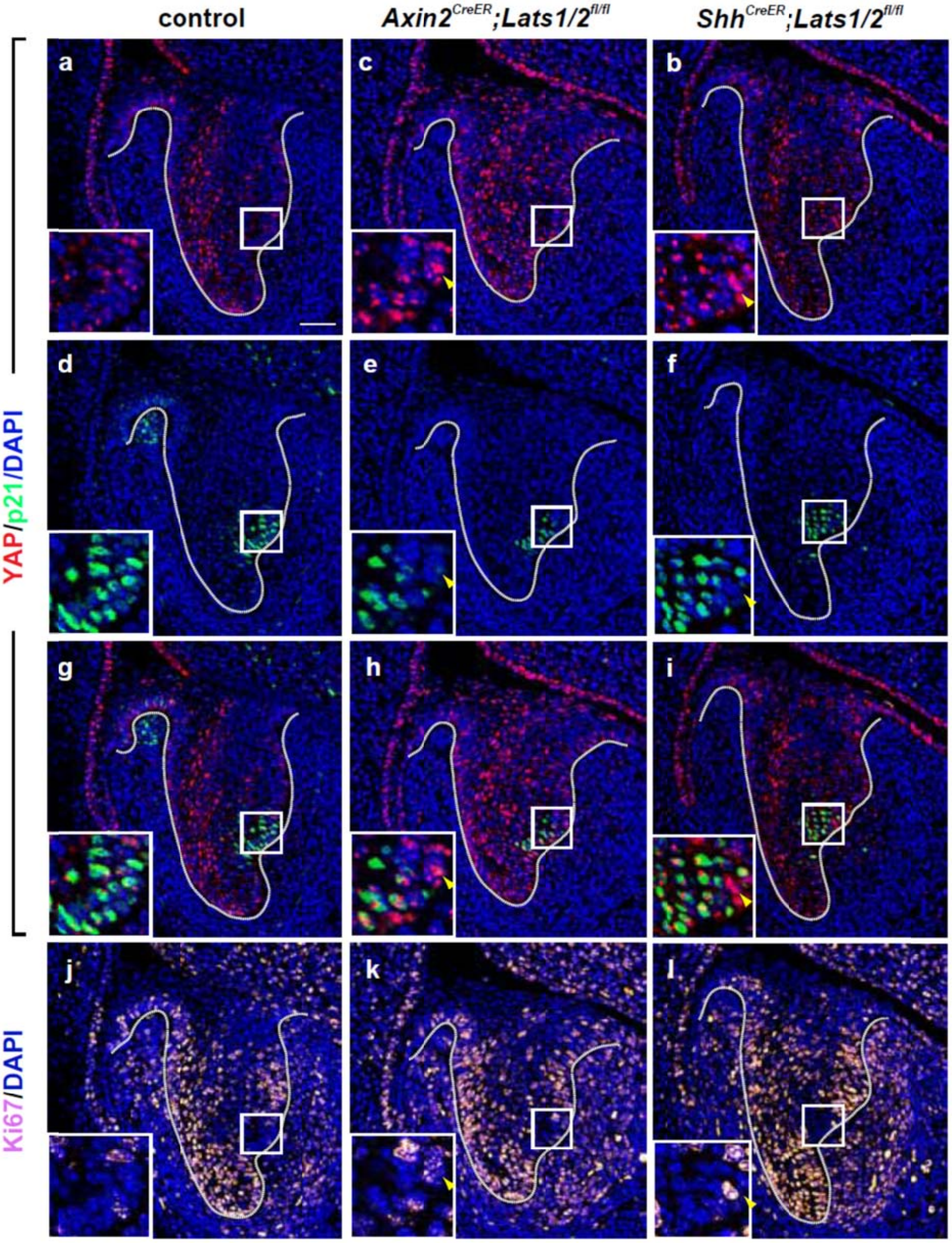

control
Axin2^{CreER};Lats1/2$^{fl/fl}$
Shh^{CreER};Lats1/2$^{fl/fl}$
YAP/p21/DAPI
Ki67/DAPI
a
c
b
d
e
f
g
h
i
j
k
l

Supplementary Figure 4: Activation of *Axin2*^{CreER} and *Shh*^{CreER} in the EK is unable to fully induce nuclear YAP at E13.5. (a,d,g,j) Nuclear YAP is excluded from the EK with increased p21 and decreased Ki67 levels at E13.5. **(b,c)** After double deletion of *Lats1* and *Lats2* in the EK, YAP begins to accumulate in the nucleus of EK cells (yellow arrowheads), although the majority of the cells exhibit low nuclear YAP. **(e,f,h,i,k,l)** p21 expression remains high and cell proliferation as assessed by Ki67 staining is unchanged after double deletion of *Lats1* and *Lats2* in the EK at E13.5. Dotted lines outline the epithelium of the incisor tooth germ. Scale bars, 50 μm in a-l.

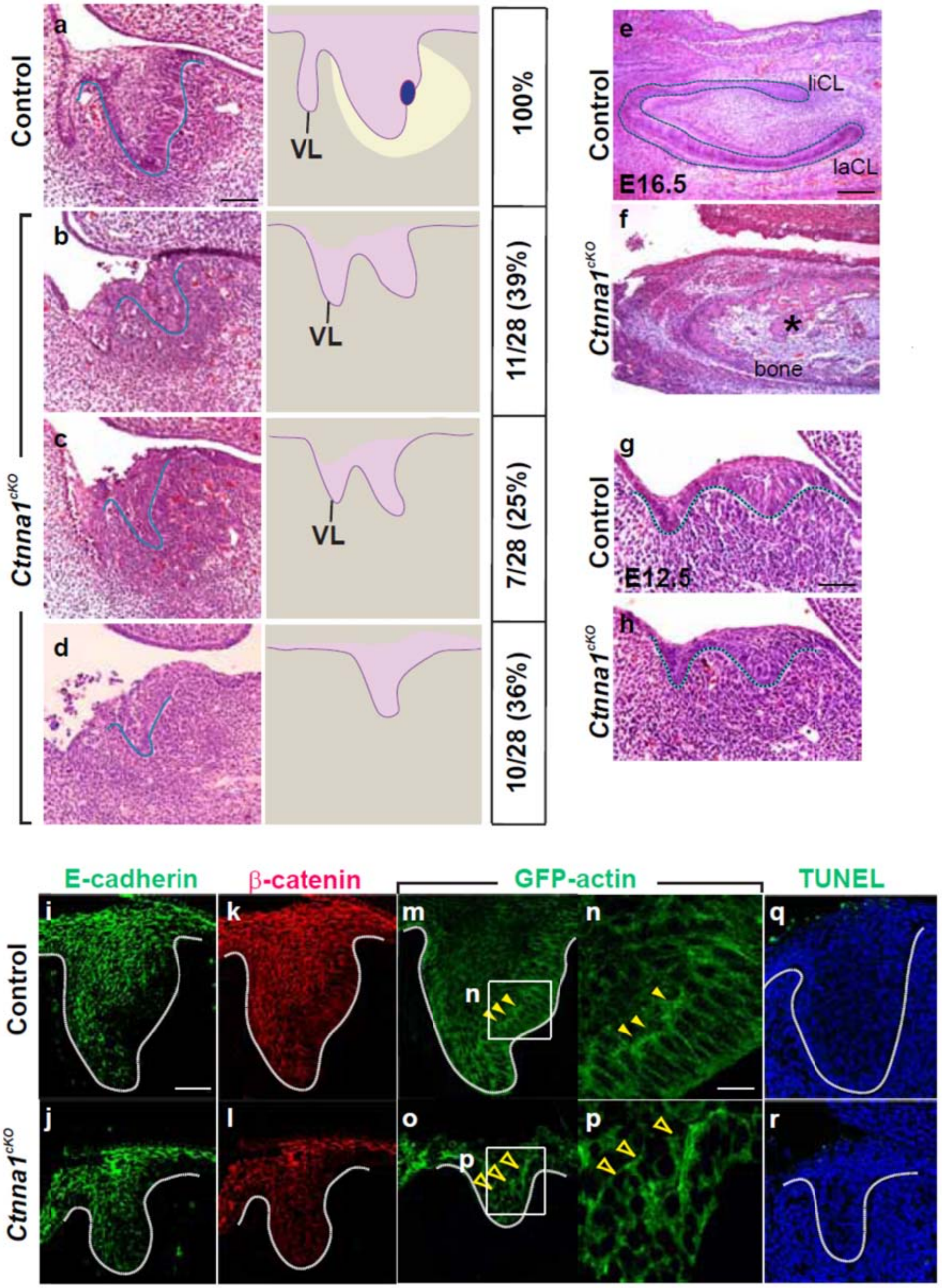

Control
a
VL
100%
b
VL
11/28 (39%)
c
VL
7/28 (25%)
Ctnna1cKO
d
10/28 (36%)
e
Control
liCL
laCL
E16.5
f
Ctnna1cKO
*
bone
g
Control
E12.5
h
Ctnna1cKO
E-cadherin
β-catenin
GFP-actin
TUNEL
i
k
m
n
q
Control
j
l
o
p
r
Ctnna1cKO

Supplementary Figure 5: Deletion of *Ctnna1* results in varied phenotypes and actin organization and constriction defects with normal expression of other adherens junction proteins. (a-d) H&E staining shows that invagination occurs in 100% of control embryos. However, in the $Ctnna1^{cKO}$, a range of abnormalities is observed (a total of 28 samples were used here). **(e,f)** H&E staining of E16.5 tooth germs shows complete loss of the developing incisor in the $Ctnna1^{cKO}$. **(g,h)** Histological analysis at E12.5 did not show obvious changes after deletion of *Ctnna1*. **(i,j)** The level of E-cadherin in the dental epithelium is increased in the EK region at E13.5 in the $Ctnna1^{cKO}$ (j) compared with control (i). **(k,l)** The level of β-catenin in the dental epithelium is not changed in the $Ctnna1^{cKO}$ at E13.5 (l) compared with control (k). **(m-p)** Actin organization and apical actin constriction (yellow and open yellow arrowheads) are disrupted at the apical side of the $Ctnna1^{cKO}$ epithelium at E13.5 (n,n') compared with that in the control (m,m'). **(q,r)** TUNEL staining at E13.5 did not show obvious changes after deletion of *Ctnna1*. Asterisk in f indicates the missing of the tooth germ at E16.5 in the $Ctnna1^{cKO}$. Dotted lines outline the epithelium of the incisor tooth germ. Scale bars, 100 µm in a-h; 25 µm in i-m,o,q,r; 12 µm in n,p.

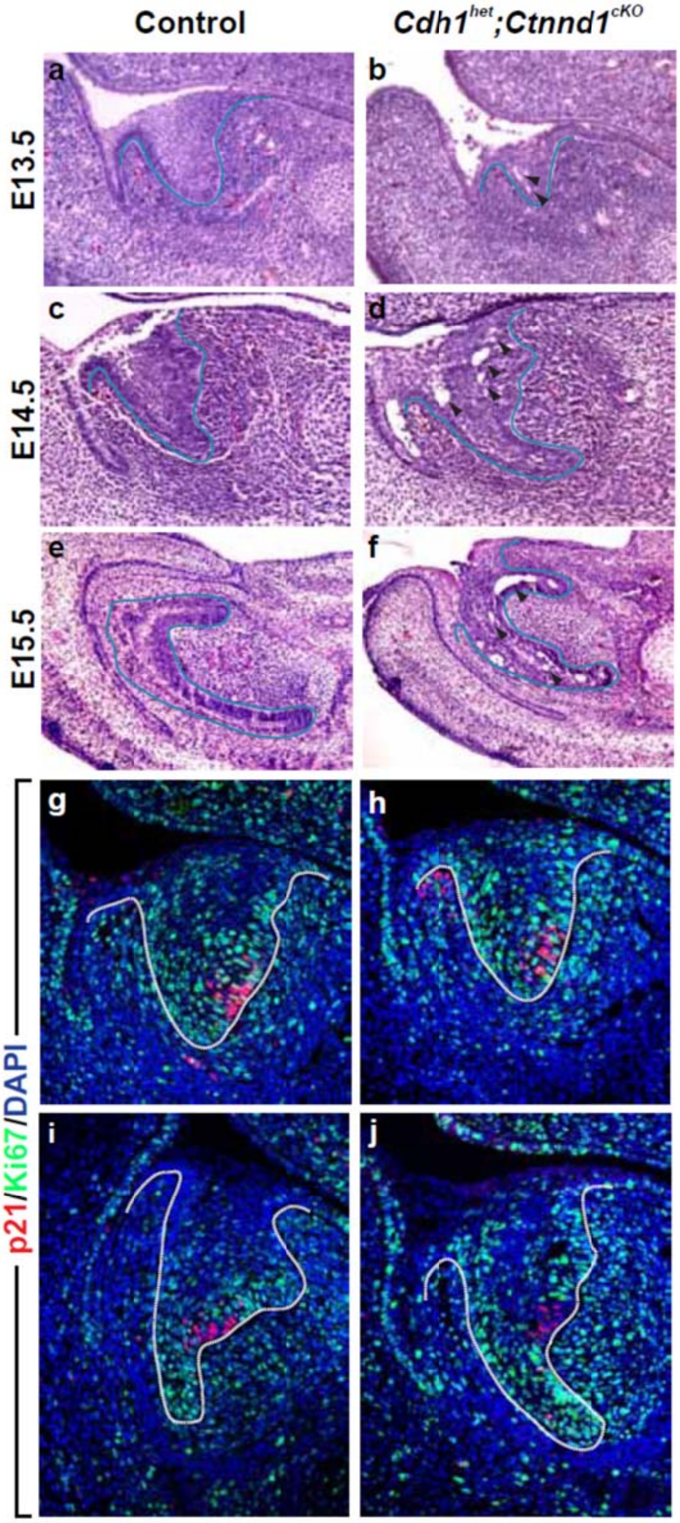
Control
Cdh1het;Ctnnd1cKO
a
b
E13.5
c
d
E14.5
e
f
E15.5
g
h
i
j
p21/Ki67/DAPI

Supplementary Figure 6: Disruption of cell-cell adhesion does not inhibit EK formation and tooth germ invagination during tooth development. (a-f) H&E staining shows that deletion of both copies of p120ctn and one copy of E-cadherin disrupts cell-cell adhesion as indicated by the gaps throughout the tissues during tooth development (dark arrowheads indicate the gaps within the tissues). **(g,h)** The EK is formed, based on the presence of p21 positive cells and ceased cell proliferation in the posterior region of the tooth germ epithelium at E13.5. **(i,j)** The formed EK is maintained throughout the cap stage, despite defective cell-cell adhesion. Dotted lines outline the epithelium of the incisor tooth germ. Scale bars, 100 µm in a-f; 50 µm in g-j.

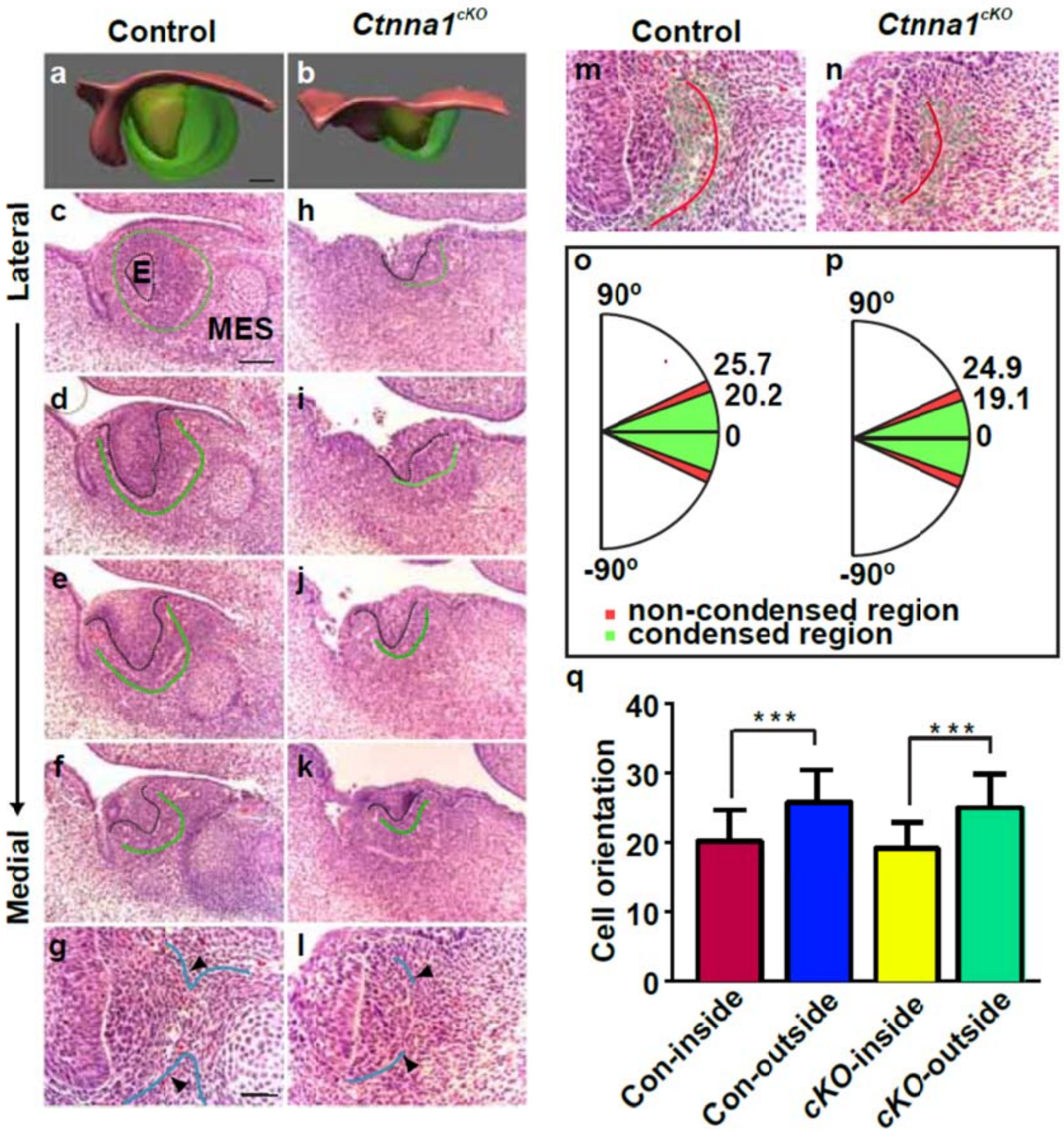

Supplementary Figure 7: 3D reconstruction of the E13.5 tooth germ and measurement of the condensed mesenchyme thickness. (a,b) 3D reconstructed E13.5 incisor tooth germs in the control and *Ctnna1*^{cKO}. **(c-f)** Lateral to medial H&E stained sections of a control tooth germ. **(g)** The border of the condensed mesenchyme in the control tooth germ. Dark arrowheads and the blue lines indicate the boarder of condensed dental mesenchyme. **(h-k)** Lateral to medical H&E stained sections of *Ctnna1*^{cKO} tooth germ. **(l)** The border of the condensed mesenchyme in the *Ctnna1*^{cKO} tooth germ. **(g-l)** High magnification shows the border of the condensed mesenchyme

in control and mutant of tooth germ. **(m-n)** Tracing the orientation of the long axis of cells in dental mesenchyme and its surrounding area marks the border of dental mesenchyme in control and *Ctnna1*^{cKO} tooth germ. **(o-p)** Radial histograms displaying quantification of dental mesenchymal cell orientation in control and *Ctnna1*^{cKO} tooth germ. **(q)** Quantification of cell orientation in dental mesenchyme and surrounding area (mean ± SEM, n=100, $p<0.001$). All quantifications are analyzed by using Student's t-test. Dark dotted lines outline the epithelium of the incisor tooth germ. Green dotted lines outline the condensed cells in the mesenchyme surrounding the epithelium. Green lines outline the long axis of cells in dental mesenchyme and surrounding area. Red lines outline the border between dental and non-dental mesenchyme based on aligned cell orientation. Scale bars, 100 μm in c-f,h-k; 50 μm in g,l,m,n.

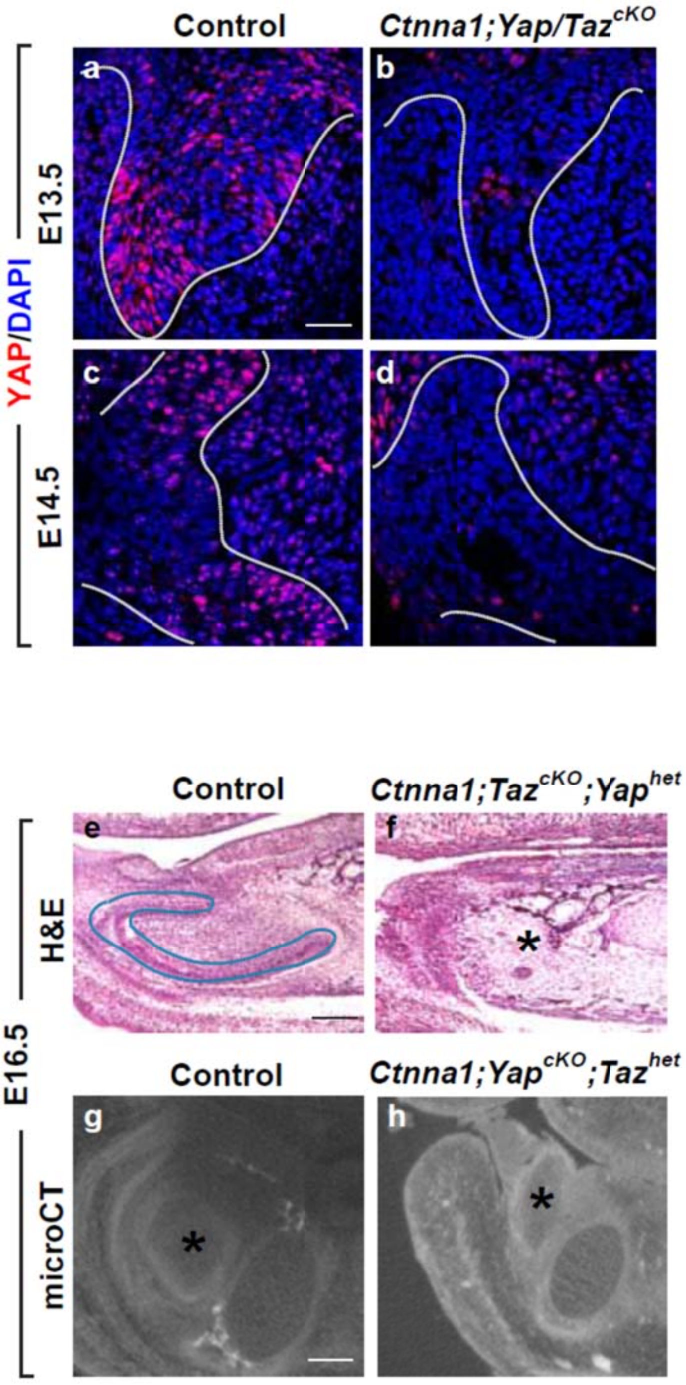

Supplementary Figure 8: αE-catenin-YAP/TAZ axis induced EK formation is required for the cervical loop invagination during late tooth development. (a-d) Immunostaining of YAP showed that YAP is mostly ablated in the tooth germ epithelium of triple mutants at E13.5 and

E14.5. **(e,f)** The tooth germ was not rescued in *K14*^{Cre}*;Ctnna1*$^{fl/fl}$*;Yap*$^{fl/+}$*;Taz*$^{fl/fl}$ mutants (asterisk). **(g,h)** The tooth germ was formed in *K14*^{Cre}*;Ctnna1*$^{fl/fl}$*;Yap*$^{fl/fl}$*;Taz*$^{fl/+}$ mutants (asterisks). Dotted lines outline the epithelium of the incisor tooth germ. Scale bars, 25 μm in a-d; 100 μm in e,f; 400 μm in g,h.

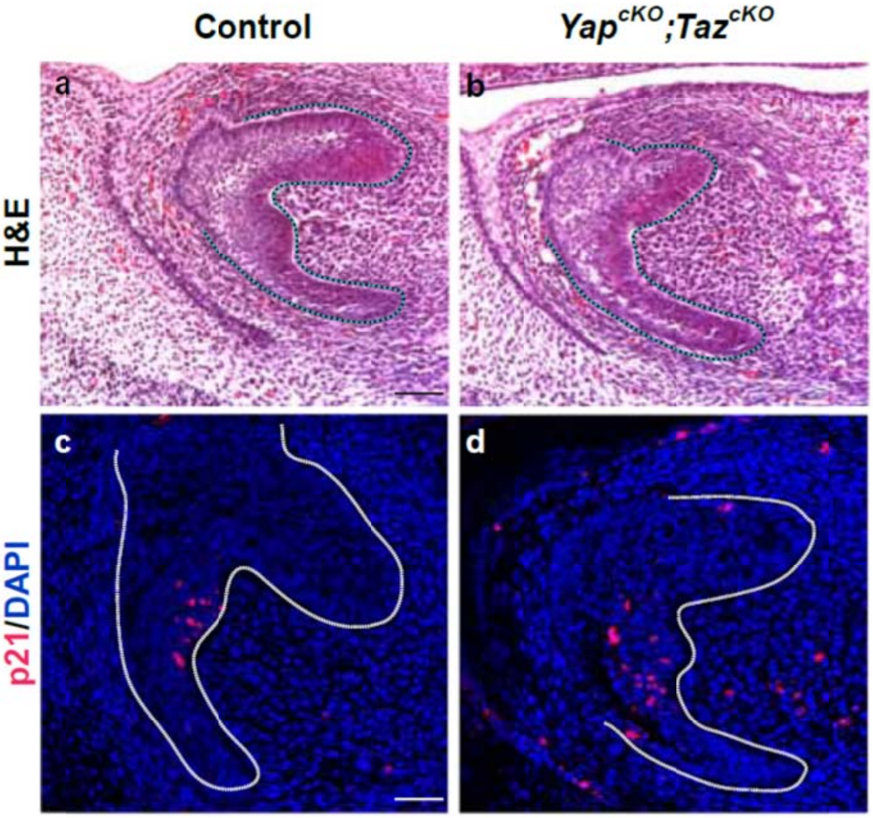

Supplementary Figure 9: Deletion of *Yap*/*Taz* in the tooth germ epithelium shows no obvious effects on tooth development. (a,b) H&E staining of E14.5 tooth germs from the control and *Yap*/*Taz* double mutants. **(c,d)** Immunofluorescence staining of p21 shows that EKs are formed both in the control and *Yap*/*Taz* mutants. Dotted lines outline the epithelium of the incisor tooth germ. Scale bars, 100 μm in a,b; 50 μm in c,d.
